# Using social networks to improve team transition prediction in professional sports

**DOI:** 10.1371/journal.pone.0268619

**Published:** 2022-06-24

**Authors:** Emily J. Evans, Rebecca Jones, Joseph Leung, Benjamin Z. Webb

**Affiliations:** Department of Mathematics, Brigham Young University, Provo, Utah, United States of America; Wroclaw University of Science and Technology, POLAND

## Abstract

We examine whether social data can be used to predict how members of Major League Baseball (MLB) and members of the National Basketball Association (NBA) transition between teams during their career. We find that incorporating social data into various machine learning algorithms substantially improves the algorithms’ ability to correctly determine these transitions in the NBA but only marginally in MLB. We also measure the extent to which player performance and team fitness data can be used to predict transitions between teams. This data, however, only slightly improves our predictions for players for both basketball and baseball players. We also consider whether social, performance, and team fitness data can be used to infer past transitions. Here we find that social data significantly improves our inference accuracy in both the NBA and MLB but player performance and team fitness data again does little to improve this score.

## 1 Introduction

Social connections exist between and across many different types of groups. This includes social relations between individuals in different schools, families, religious and professional groups, or any other group defined by affiliation. An important feature of these social connections is that they are not always between members of the same group.

In this paper, we address how these relations affect individual transitions between groups. The social connections we study are those formed between members of professional sports teams, specifically the social connections between the players in the MLB (Major League Baseball) and the players in the NBA (National Basketball Association), respectively. The transitions we study are the player transitions from one team to another within MLB and the NBA, respectively, during a player’s career.

These baseball and basketball teams can be thought of as specific types of *professional groups*, i.e., groups of individuals employed by the same employer, with a similar skill set, and a specific objective. Transitions between such professional groups are not the same as transitions between *social communities*, which are communities defined purely in terms of social interactions [1]. The dynamics of individual, or more generally, node transition between social communities within social networks is a relatively new field taking cues from mathematics [2] and computer science [3]. As this is not the focus of this paper, we refer the interested reader to a survey of work [4].

Transitions between professional groups have been previously studied by sociologists and economists (see for example [5–13]). To understand these transitions, features such as the strength of ties between workers [5], the geography of transitions [7], the role social networks play in finding employers and employees [6, 8] are considered. In regards to the social network aspect of such transitions, specific questions that have been considered include whether companies hire workers through social connections of productive employees [9], whether employees hired through referrals are more likely to stay [11], how unemployed workers find jobs through their social networks [10, 13], differences in salaries between employees found by referrals [6], and motivations for employees to grow their professional social networks [12].

Since social connections often exist between individuals in different professional groups, a natural question is how these connections influence transitions between such groups. Here we consider the specific question of how social data, player performance, and team statistics can be used to improve our ability to predict the way professional athletes transition from one professional group to another, i.e., one team to another specifically in the MLB and NBA.

Other ways of analyzing team transitions in the NBA and MLB that have been considered include analyzing the labor market’s influence on professional baseball and basketball players, which has been studied extensively beginning with the classic work of Rottenberg [14]. The transition between teams in baseball has been studied more recently in light of changes to rules governing transitions [15] and also in terms of player productivity [16]. In professional basketball, hiring decisions have been considered with regards to first-hand experience [17] and also in terms of increased productivity [18]. Tools from network theory have also been used to study interactions in both baseball [19] and basketball [20, 21]. However none of the listed works consider how a player’s *social-professional network* influences transitions between teams despite the fact that social network analysis has become increasingly popular in sports analytics [22].

From the various professional groups that exist, a major factor in choosing to analyze team dynamics of the MLB and NBA is the availability of data. This includes the player’s social data but also information such as the player’s performance, and other factors that could be used to predict transitions between teams. The size of the data set, measured in terms of the number of individuals, the number of years it spans, and variety of statistics is also important as our analysis relies on machine learning algorithms that require sufficient amounts of data to both decrease bias and improve accuracy (see Section 4 and [23]).

To address the question of what influences transitions between professional teams we consider three factors: individual performance, team fitness, and social data. Of the three, *individual performance* is perhaps the most natural to consider. The idea is that poor performance presumably motivates managers to replace players while high performance makes players more attractive to other teams. To understand this tendency of professional athlete’s to move from one team to another, we also considered team *fitness* together with *individual performance*. Here the idea is that an athlete with high performance is more likely to transition to a team he or she perceives as either being fit, or becoming more fit [24]. A natural assumption is that an athlete with low performance is more likely to get traded to a team with lower fitness, which can help in predicting transitions.

In the context of group dynamics there are many ways to measure *fitness* including how cohesive or stable the group is [25], the strength of individual members, and the ability of the group to perform its designated task. In this study, we considered two proxies which we use to measure the fitness of our groups. The first is *relative team ranking*, which acts as a measure of a team’s ability to achieve success. The second proxy for team fitness is the *financial valuation* of a team, which is based on the notion that a team on firm financial footing is more stable and can likely offer high performers more competitive salaries [26].

The third factor we consider is the *social interactions* individuals have within their social-professional network (see Section 3.4). The idea here is that, if the player has social connections to other players from other teams, then this may indicate at least a predisposition to move to that team when compared to other teams.

Our study considers two proxies for the players’ social-professional networks. The first proxy is a snapshot of the Twitter connections that existed between players in 2019. This data set describes which players followed which players up to when this data was collected. The second proxy of a player’s social-professional network is created using the college the player attended, which we refer to as the player’s *college network*. This was collected players in the NBA and was done to test whether a shared collegiate experience has an effect on the transitions players make throughout their career.

As the Twitter data is not retro-actively timestamped it can only be used to predict player transitions after it was collected. However, it can be used to infer transitions that occurred prior to 2019. Our first goal is to understand how well this data can be used to *predict* player transitions for the 2020–2021 seasons then to use this data to *infer* transitions prior to and including 2019. The difference between what we can infer and what we can predict gives us a sense of the changes in players’ social-professional activity on Twitter before and after transitions.

Similarly we use the NBA players’ college network to predict future player transitions. Our goal here with using this college data, similar to using Twitter data, is to understand how well this data predicts future transitions when it is used with and without performance and fitness data. The overarching question we hope to answer is how different combination of these three factors– *individual performance*, *team fitness*, and *social interaction*– improves or decreases our ability to infer and predict to which team an individual will transition to.

What we find is that the use of Twitter data significantly improves our ability to predict transitions for players in the NBA but does little to change our accuracy in predicting MLB transitions. Similarly, the addition of performance and team data only slightly change our prediction accuracy in both the NBA and MLB from that of a random guess (see Section 5.1). Including the college a player attended, which is our second proxy for social data in the NBA, similarly increases our prediction accuracy nearly as much as Twitter data. Overall this suggests that social connections are much more important in the NBA than MLB in predicting the destination of player transitions.

For inferring past transitions the addition of performance data, team fitness data, and social data each improve the accuracy of the machine learning algorithms we consider for both the players in MLB and in the NBA. Performance and team fitness, perhaps surprisingly, only modestly raise the accuracy of our results. The inclusion of social data from Twitter, however, dramatically improves the predictive ability of these algorithms when predicting past transitions in every case we consider. Here predictions are typically better for the NBA than for MLB. This again suggests that social connections are less important in MLB than in the NBA. (See Section 2 for a summary of these results).

**Fig 1:**
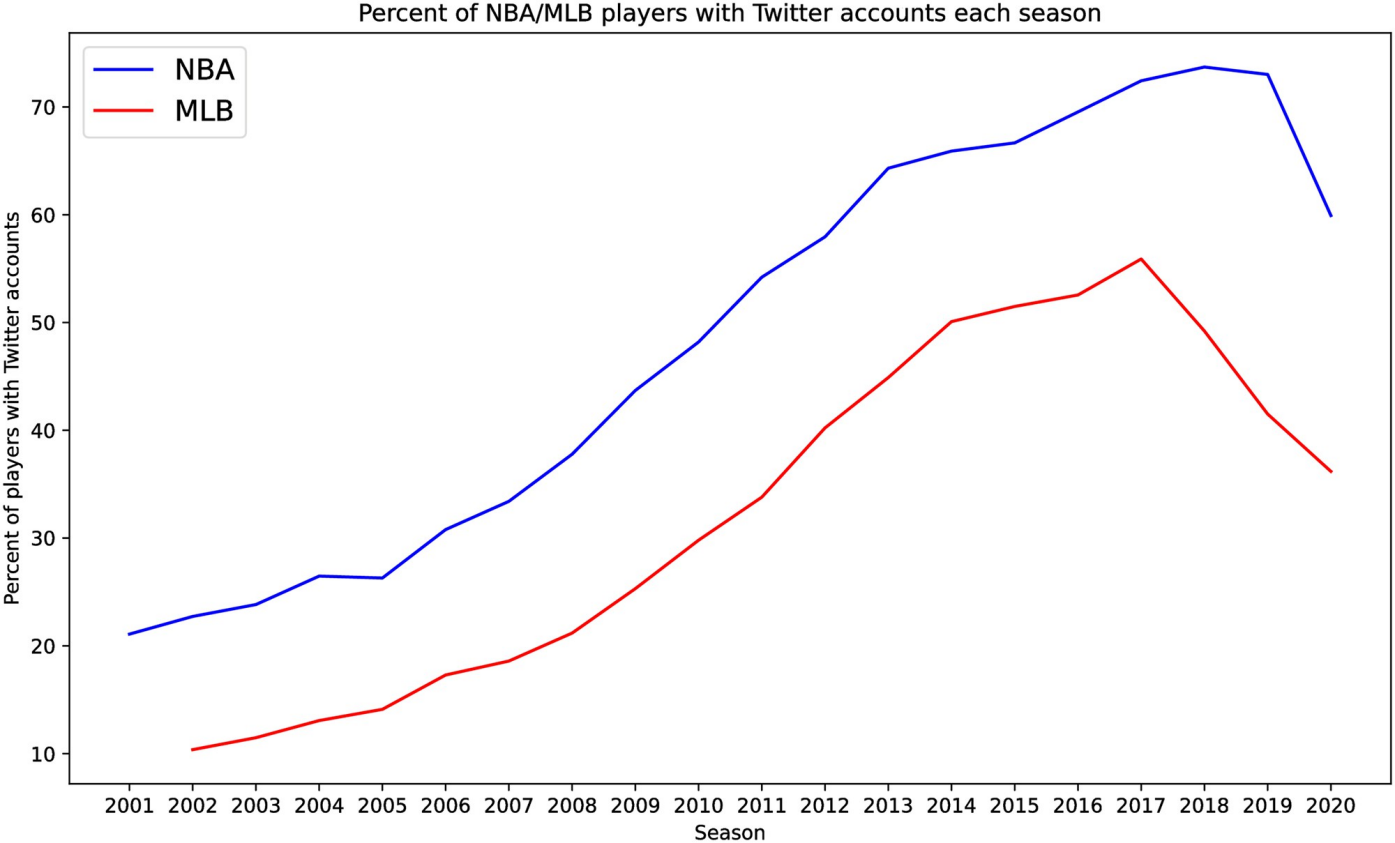
A comparison of the percentage of players who had an active Twitter account before and including July 2020 and who played between 2002—2018 for the MLB (blue) and between 2001—2019 for the NBA (orange). The datasets we use only give the number of players that have Twitter and who played in a given year, not the specific players that used Twitter during that year. This explains why there is Twitter data as early as 2001, even though Twitter began in 2006.

An interesting feature of the Twitter data is that, over time, an increasing number of players in both the MLB and NBA begin to follow other players (see Fig 1). When we limit our methods to the latter decade of our study when Twitter use is at its highest, we can infer transitions much more accurately for both MLB and the NBA than for the first decade (see Tables 11 and 12).

We also find that although the Twitter networks for baseball and basketball are fairly different in size, the two networks are strikingly similar. Specifically, they have very similar network statistics including mean degree, fraction of nodes in the largest strongly connected component, mean distance between connected node pairs, clustering coefficient, reciprocity, and the degree assortativity (see Table 13). Therefore, it seems unlikely that the particular structure of these networks can explain why Twitter data leads to higher prediction and inference accuracy for basketball when compared to baseball.

The paper is organized as follows. In Section 2 we give a brief summary of our results regarding prediction and inference accuracy in both MLB and the NBA. In Section 3 we describe our methodology including which social and nonsocial data we collected and some of the features of this data. This includes performance, fitness, social, and other data we used to train the machine learning algorithms we selected. In Section 4 we give a brief description of these algorithms. In Section 5 we describe how different combinations of social and nonsocial data effect the accuracy of these algorithms. In Section 6 we analyze the basic statistics of the baseball and basketball Twitter networks. In Section 7 we discuss a few limitations of our data and methods of analysis. We conclude in Section 8 with some open questions that specifically relate to how this type of analysis could be extended to study group transitions in other settings, i.e., other professional groups and more general social networks.

## 2 Summary of results

Here we give a brief summary of the results found in Section 5 regarding the accuracy of the machine learning algorithms we consider. The different types of data we use to determine player transition between teams are broadly speaking *player performance*, *team fitness*, and *social data*, which are described in detail in Section 3.

When predicting future transitions in both the NBA and MLB we find that the addition of player and team data does little to raise our prediction accuracy over the probability 1/29 ≈ 3.45% of a correct random guess. In fact, using all nonsocial data improves our accuracy by at most 1% over this probability. In contrast, using social data dramatically improves our accuracy in predicting transitions in the NBA. Using Twitter data alone allows for an accuracy of up to 20% while using college data gives us an accuracy of up to 17.4% with similar F1 scores. Using social data to predict transition in MLB, however, has little effect, only slightly improving scores beyond the probablity of a correct guess. In fact, the inclusion of social data alone only gives an accuracy of 4.6% which is not as good as only taking into account the career length of the player which gives an accuracy of 6.7%. Here the inclusion of social data typically *decreases* the accuracy of our machine learning algorithms for baseball leading us to conclude that social data does not provide any valuable information as far as transitions are concerned. (See Tables 7 and 8).

We find when inferring past transition that the addition of each of player performance, team fitness, and social data each improve the predictive ability the algorithms we consider. However, as mentioned performance data by itself does little to raise our inference and prediction accuracy. Specifically, including performance data raises the accuracy of these algorithms by at most 1% for both the MLB and the NBA over the probability of a correct guess. Similarly, using team fitness data improves accuracy by at most 0.85% for the MLB and 1.35% for the NBA. Using all nonsocial data together including performance, team fitness, player position, team, and career length improves accuracy by at most 1.055% for the MLB and 5.25% for the NBA (see Tables 9 and 10).

When using social data to infer past transitions the situation improves significantly. When using data derived from Twitter connections, with no other information, the prediction accuracy of the algorithms can be as high as 21.2% for the MLB and 27.4% for the NBA, an increase of 17.75% and 23.95% over random guessing, respectively. Using college data for the NBA similarly increases the accuracy of prediction to as much as 8.8%. The F1 scores follow the same pattern. It is worth noting that our maximum accuracy is found in the MLB using only the player’s team together with Twitter data while in the NBA our maximum accuracy is found using only the team’s fitness combined with Twitter data (see Tables 9 and 10).

As mentioned in the introduction, over time an increasing number of players in both the MLB and NBA to follow other players (see Fig 1). When we limit our predictions to the last decade of our study when Twitter use is at its highest, we can predict with up to 19.4% of the time where a player will transition to in the MLB and up to 30.2% of the time in the NBA (see Tables 11 and 12).

## 3 Methodology

### 3.1 Data collection

Performance data was scraped from www.basketball-reference.com/leagues and www.baseball-reference.com/leagues using Python and Beautiful Soup both of which are packages used to extract data from htmls. Since we looked at historical data and used appropriate crawl delays, we met the scraping terms defined in the robots .txt file for both sites. The Twitter data was collected using the Twitter API. First, we scraped the Twitter usernames for each player listed on www.baseball-reference.com/friv/baseball-player-twitter-accounts.shtml and www.basketball-reference.com/friv/twitter.html. Then using **tweepy**, a python package for connecting to the Twitter API, we were able to collect the Twitter IDs of the other MLB/NBA players that each player followed. We chose to look at those “followed” instead of those “following” because it significantly sped up the data collection process. By using the Twitter API and tweepy, we were able to follow all necessary protocols, including rate limits and only accessing publicly available information. The data is publicly available at https://doi.org/10.5061/dryad.g4f4qrfs5.

As all data used in the development of our datasets came from publicly available websites and included only factual data about people, IRB approval was not required. In addition we anonymized the data both in the paper and in the datasets available on https://doi.org/10.5061/dryad.g4f4qrfs5.

### 3.2 Baseball performance dataset

Major League Baseball consists of 30 teams evenly split between the American and the National league. Each full team roster consists of 40 players. A baseball season consists of 162 regular season games with some players in certain positions playing most games, and some players in positions like pitcher playing in a fraction of these games. In our analysis we consider 3 high-level positions that players can be in: pitcher, catcher, and fielder, where the position of *fielder* represents all other positions. We note that positions are more fine grained, but typically players who play in the infield and outfield have some flexibility in the actual position that they play. We singled out the catcher position because, usually, one of the catchers serves as team captain. It is worth noting that the exact composition of a team’s roster varies with some teams having more of one position than another. The baseball performance data for the 2002—2021 seasons we use were obtained from https://www.baseball-reference.com. Although the website contains a wide variety of statistics such as number of games played, points scored, and total hits for our analysis we focused primarily on a few advanced statistics and a few engineered statistics instead of generic totals. The data collected for a player includes the main position played, the team played on, and the player’s age for a given season. The advanced data we collected for each player includes: the *fielding percentage* (FLD%), *offensive winning percentage* (OWn%), *adjusted batting runs* (BtRuns), and *adjusted batting wins* (BtWins).

OWn% is the percentage of games that a team would win if the batting was done by 9 copies of the player, assuming average offense and defense. BtWins estimates a player’s total contribution to his team’s wins with his bats. BtRuns is an estimate of a player’s running contribution to a team’s wins. FLD is the number of putouts and assists divided by the sum of putouts, assists, and fielding errors. This data provides an overall picture of a player’s performance during the season. While other metrics are often used in evaluating player performance, we selected metrics that were representative of both pitching and catching positions and were available on www.baseball-reference.com.

We then created the following engineered data for each player and each season:

- Position—created by merging actual players positions into the three positions we identified: pitcher, catcher, and fielder.
- Career length—number of prior seasons played until the year under consideration (i.e., rookies have a career length of zero).
- Leave variable—specifies if a player is to leave their current team after the season under consideration.
- Target variable—specifies which team a player plays for the next year, or if they do not return to play that next year.

The *leave variable* is critical in identifying which players transition at the end of the season to another team allowing us to focus on predicting the transitions of only those players. The *target variable* provides us the ground truth for measuring the accuracy of our results.

To illustrate our collected and engineered features we display three seasons of data for a random baseball player in Table 1. We note that at the end of 2017, this player switched teams, (to the New York Yankees), hence the engineered field of target was set to NYY.

**Table 1:** Three years of collected and feature engineered data for a random baseball player. We observe that at the conclusion of the 2017 season, this player transitioned from the Miami Marlins (MIA) to the New York Yankees (NYY). Thus in 2017 his *target* value is set to NYY. In this table, and in Table 3 we use the abbreviation CL for career length (an engineered variable).

Season	Position	Team	FLD%	OWn%	BtWins	BtRuns	CL	Target
2016	FD	MIA	.982	1.2	.585	11.9	7	N/A
2017	FD	MIA	.998	.735	59.8	5.6	8	NYY
2018	FD	NYY	.992	.621	26.7	2.6	9	N/A

We show the specific distribution of players, players leaving their team, players retiring, and players transitioning for each year in Table 2. We note that each year approximately 50% of players leave their team in some manner.

**Table 2:** The number of Major League baseball players per year comprising the 2002–2020 baseball seasons. We observe that each year 48.7% of the players leave their current team on average. Of those that transition about one-half, 49.0%, of the players transition to a new team and the other half of the players end their professional careers.

Season	2002	2003	2004	2005	2006	2007	
# of Players	1090	1124	1125	1127	1116	1162	
Total Leaving	527	606	560	605	513	566	
Retiring	236	274	276	283	257	302	
Switched Teams	291	332	284	322	256	254	
Season	2008	2009	2010	2011	2012	2013	
# of Players	1166	1142	1151	1160	1176	1194	
Total Leaving	565	531	538	551	565	562	
Retiring	288	281	287	271	273	281	
Switched Teams	277	250	251	280	292	281	
Season	2014	2015	2016	2017	2018	2019	2020
# of Players	1204	1243	1233	1222	1266	1407	1285
Total Leaving	610	633	585	578	601	696	621
Retiring	279	321	326	288	285	451	365
Switched Teams	331	312	259	290	316	245	256

### 3.3 Basketball performance dataset

Similar to baseball, the National Basketball Association consists of 30 teams evenly split between two conferences. In the NBA, each team’s roster consists of only 17 players, with only eight players required to be active at any one time. Basketball has five positions: point guard, shooting guard, small forward, power forward, and center; however most basketball players are capable of playing in more than one of the positions. Each team plays 82 games in a standard season.

The basketball performance data for the 2001–2021 seasons was collected from www.basketball-reference.com. Similar to baseball we choose to use advanced data statistics, focusing on three advanced stats. PER, *Player Efficiency Rating*, measures how much a player produced in one minute of play. *Win Shares* or WS is an estimate of how many wins were contributed by a player. BPM, *Box Plus/Minus*, is an estimate of the number of points per 100 possessions that a player contributed. To illustrate both the collected and engineered statistics we consider a few seasons of a representative basketball player’s career in Table 3, and note that he switched teams in 2018.

**Table 3:** Three years of collected and engineered data for our representative basketball player. At the conclusion of the 2018 season, this player transitioned from the New Orleans Pelicans (NOP) to the Golden State Warriors (GSW) so the target variable was set to be GSW.

Season	Team	PER	WS	BPM	CL	Target
2017	NOP	23.2	1.6	5.5	6	N/A
2018	NOP	22.6	4.7	4.7	7	GSW
2019	GSW	21.4	2.4	3	8	N/A

Similar to baseball, we also created engineered features for individuals each season. Since basketball has only 5 positions, we did not modify this feature, and only engineered values for career length, leave and target. Ultimately, there were 3688 basketball players who switched teams between 2001 and 2020. The distribution of the leaving players is shown in Table 4. The average percent of players leaving their team each year is 67%, and approximately 32% of those that leave retire.

**Table 4:** The number of National Basketball Association (NBA) players per year comprising the 2001–2020 seasons. We note that there are approximately half the number of players in this dataset compared to the baseball dataset. Also, a higher percentage of players transition annually in the NBA (on average about 58%). A high amount of players switch teams each year, about 67%, while only 32% retire.

Season	2001	2002	2003	2004	2005	2006	2007
# of Players	441	440	428	442	464	458	458
Total Leaving	251	238	284	289	282	245	253
Retiring	84	43	85	78	62	98	89
Switched Teams	167	153	206	227	184	156	166
Season	2008	2009	2010	2011	2012	2013	
# of Players	450	444	442	452	478	468	
Total Leaving	258	259	281	257	295	273	
Retiring	85	79	71	73	100	83	
Switched Teams	173	180	210	184	195	190	
Season	2014	2015	2016	2017	2018	2019	2020
# of Players	481	492	476	486	540	530	529
Total Leaving	267	279	259	270	310	334	296
Retiring	86	96	90	94	128	130	94
Switched Teams	181	183	169	176	182	204	202

### 3.4 Social network datasets

As it is extremely difficult to impossible to create a ground truth social-professional network for players, we created an approximation of this network utilizing Twitter data. Twitter is a social networking site that allows users to exchange short “Tweets” with followers. Twitter was chosen because player Twitter handles were available from both www.basketball-reference.com and www.baseball-reference.com, and because Twitter provides an easy API that can be used to obtain both the followers and those followed by a user. A downside of using Twitter is that the “followers” information is not time stamped. Hence our network created with the Twitter data is a snap shot of the relationships that existed before and up to July 2020 when we scraped the data with no way to pinpoint when a player started to follow another.

**Fig 2:**
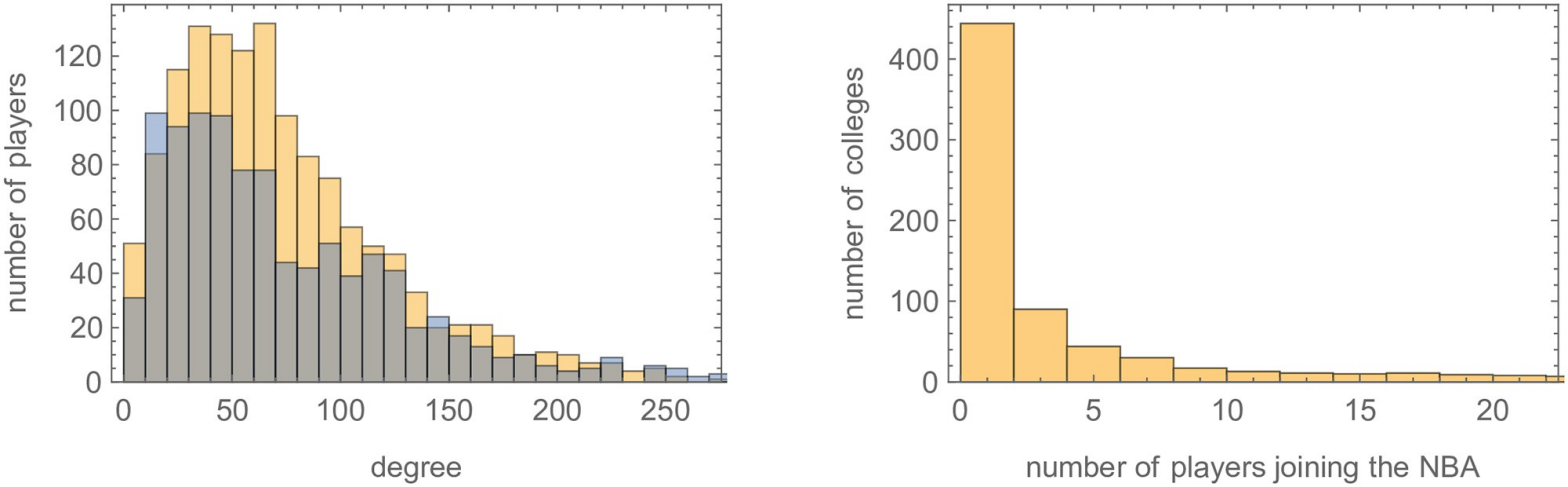
Left: A histogram of the degree (centrality) for the baseball Twitter network and the basketball Twitter network shown in orange and grey, respectively. Right: A histogram of the number of colleges that had one of more basketball players join the NBA from 2001–2019.

With the Twitter data we created a directed social network of players where player A has a connection directed to Player B if Player A followed Player B, which we refer to as our *baseball Twitter network* and *basketball Twitter network*, respectively. Of the 4207 unique baseball players that switched to a different team from 2002—2018, we were able to obtain Twitter handles for 702 of them. For basketball players that switched between 2001—2019, we were able to collect Twitter handles for 784 of the 1847 players, a significantly larger percentage indicating how active NBA players are on Twitter compared with MLB player (see Fig 1 and Table 5). The resulting Twitter network is a social network with 53690 directed edges for baseball and 43827 directed edges for basketball. Most players in both datasets have a relatively small number of connections or *degree (centrality)* to others, which is the number of followers together with number of players followed for a specific player. A few players do have a large number of connections though (over 100). The distribution of connections for both baseball and basketball players having at least one Twitter connection is shown in Fig 2 (left). (A more thorough analysis of these networks is given in Section 6).

**Table 5:** The number of MLB and NBA players in the final dataset, which contains those players who switched to a different team and had a Twitter account.

Season	2001	2002	2003	2004	2005	2006	2007	2008	2009	2010
MLB	NA	23	31	30	48	43	39	62	61	69
NBA	41	31	48	60	50	43	58	73	82	123
Season	2011	2012	2013	2014	2015	2016	2017	2018	2019	2020
MLB	94	119	123	151	148	138	157	169	103	111
NBA	121	127	141	150	145	128	150	148	180	153

For the NBA we also investigate whether the college a player attended can serve as a proxy for social connections and whether this data helps predict where player’s transfer during their professional career. To test this idea, we pulled the college data for each basketball player from www.basketball-reference.com and created a categorical feature for colleges. If a player in this set did not go to college, they were included and their college category was *N/A*.

In total there were 259 different colleges attended by future NBA players in the data set. Fig 4 (right) shows the number of colleges in the data set that had a given number of players attend. The *N/A* category is excluded. For example, from the data there were 5 schools that each had between 15 and 17 players attend: Syracuse: 16, Michigan State: 15, Georgetown: 17, LSU: 15, and Georgia Tech: 16. 940 players did not attend a college. More complete statistics are found in Table 29 in S1 Appendix.

**Fig 3:**
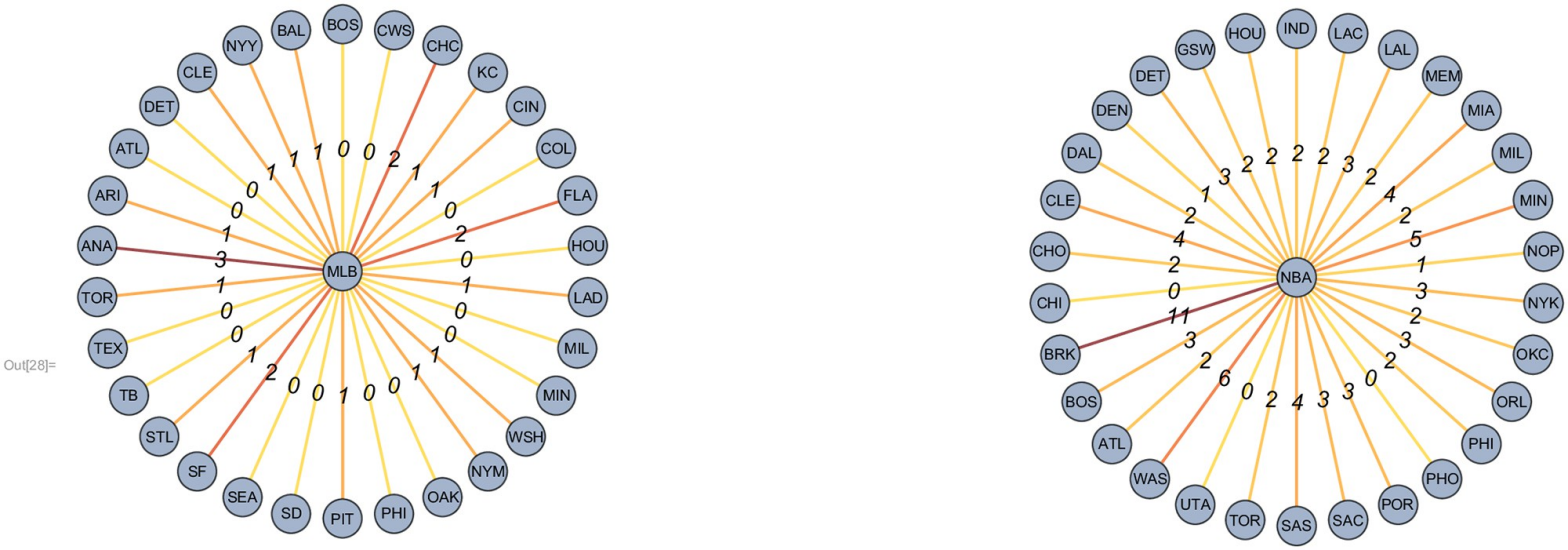
Left: The affinity network of a representative MLB player from the 2017 season. Right: The affinity network of a representative NBA player from the 2018 season. In both, edge weights indicate the player’s affinity score where darker edge colors indicate a higher score.

Using our Twitter networks and the team each player played on for a given season we create an *affinity network* for each player as follows: For a given transitioning player we add a weighted edge connecting the player to each of the teams in the MLB/NBA. The weight of an edge is the number of other players from that team this player *followed* during that season, which we call the *affinity score* (see Fig 3). We emphasize that the social network between players is fixed across seasons but the social affinity score *changes* between seasons since players change teams. This weight gives a score of the affinity that a player has for the team for a given season. Since we do not allow a player who has been identified as *transitioning* to remain on their current team we set the affinity score for the current team to zero. Finally the way we handle mid-year transitions (i.e., midyear trades) is different between the two sports. In basketball we consider only the team the player was on at the beginning of the season. For baseball, due to the way information is presented at baseball-reference.com we omit players who transitioned during mid-season from the calculation of the affinity score for a given year.

### 3.5 Team stratification engineered data

Using the idea that successful players move to successful teams, at least on average, we created a measure of team fitness. We collected data on each team’s dollar valuation for each year in question through Forbes.com. We also retrieved team rankings for each year from www.basketball-reference.com and www.baseball-reference.com The result is a number of new features in each of the players’ data (see Table 6 which extends Table 3).

**Table 6:** Three years of collected team data for a representative basketball player.

Season	Team	Rank	Value	Target
2017	NOP	20	750	N/A
2018	NOP	8	1000	GSW
2019	GSW	2	3500	N/A

## 4 Analysis techniques

In this section, we describe the techniques used to *make* our group transition predictions. We chose to utilize machine learning methods, rather than more classical statistical techniques, to see if different metrics, rather than those traditionally used, could provide better predictive power. In a nod to more traditional techniques we include logistic regression for comparison. We did not include neural networks or deep learning algorithms because our data set was not rich enough support the data requirements of these algorithms and typical data augmentation techniques are not easily applicable to the problem at hand.

Since our question was a classification question, the majority of the techniques we use are ensemble methods. Ensemble methods combine several models (predictors) which operate independently and are typically good for classification problems. We use four types of ensemble methods to make predictions which can be classified into two different categories: (i) *Randomized decision trees* which include (a) Random Forests and (b) Extremely Randomized Trees, and (ii) *boosting algorithms* which include (c) Adaptive Boosting and (d) Extreme Gradient Boosting. As mentioned, we also use (e) Logistic Regression, a more traditional technique and (f) *k*-Nearest Neighbors as these are also commonly used for classification.

### 4.1 Random decision trees

The idea behind randomized decision trees is rooted in the construction of a classification tree. A classification tree takes input data, moves through the various decisions nodes of the tree to a leaf, and returns as output the most common result in that leaf. At each level of a classification tree a decision node is constructed by considering the features not already split on, choosing the best feature to split on, and then choosing the optimal split point [23]. Random decision trees randomize the creation of classification trees in two ways. The first is that a random subset of the data is sampled and from that subset a classification tree is created. A standard classification tree considers all of the remaining features when deciding which feature to split on. However, this often results in trees that are highly correlated. Instead of using all the remaining features at each level of the decision tree, a random decision tree also chooses a random subset of the remaining features to split on. This offers two advantages, the first is a collection of uncorrelated trees, and the second is splitting on fewer features results in faster algorithms.

The random forests algorithm (Forest) operates by having many decision trees, which are trained on different random parts of the training set, “vote” on the final classification [27]. A typical random forest consists of thousands of these voting decision trees, and it is typically the case that some of them are actually good models. Random forests rely on Condorcet’s Jury Theorem from political science which guarantees a collection of weak voters will arrive at the correct decision with high probability [28].

In the extremely randomized trees (ExtraTrees) algorithm, instead of looking for an optimal splitting threshold for each feature at each step of the decision tree, thresholds are created at random. The splitting rule for each tree is chosen to be the best of these random thresholds. This results in trees that are created more quickly and with lower variance in the model at the cost of a slight increase in bias. These trees similarly “vote” as in the case of the random forests algorithm.

### 4.2 Boosting algorithms

Boosting algorithms are a family of algorithms whose aim is to create a strong learner from a weak learner. Boosting algorithms work by applying the weak learner sequentially to weighted versions of the data where in each sequential application misclassified data is given additional weight. The weak learner can be any classification or regression model, but the most frequently used learner is a decision tree [23]. Important to the construction of a boosted tree is the choice of a loss function, which measures the predictive error of the model. The goal of boosting is to minimize this loss function, and this is done sequentially using the idea of gradient descent.

In our work we consider two different boosting algorithms that use decision trees as learners: Adaptive Boosting (AdaBoost) and eXtreme Gradient Boosting (XGBoost). Adaboost [29] was the original boosting algorithm and has the characteristic that the decision trees have a single split, sometimes called decision stumps. XGBoost [30] is a more recent algorithm for boosting and combines decision trees with more splits and sophisticated algorithms to improve the time it takes for the algorithm to converge to the optimal tree. XGBoost is extremely popular due to its ease in configuring, its relative speed in running and its high accuracy.

### 4.3 Other algorithms

In addition to ensemble methods we also use two other classification methods. The first is multinomial logistic regression (softmax regression) which uses a combination of the softmax function and the technique of regression to construct a multi-class classifier [23]. In the context of our problem, multinomial Logistic Regression (LogReg) returns a probability vector where each entry in the vector is a probability that the player transitions to a given team. The second classification technique we use is that of *k*-nearest neighbors (KNN) [31]. In this technique *k* nearest neighbors are chosen and a probability of a player transitioning to a given team is the proportion of those neighbors that belong to the given team.

### 4.4 Tools and methods

The tests were run using Python, Pandas, and Scikit-learn. After collecting the data, we use Pandas, a Python database package, to create the final data sets. Scikit-learn is a widely used Python package for machine learning. All of the algorithms except XGBoost were implemented using Scikit-learn algorithms. XGBoost was implemented using xgboost, a package for running XGBoost in several popular languages.

SkLearn’s GridSearch was performed to identify appropriate hyperparameters. Since the MLB and NBA data sets were different, the search was performed separately on both data sets. The values for the hyperparameters used can be found in the Table 30 in S1 Appendix. For more information on the hyperparameters, see the sklearn documentation for each algorithm.

Since it was possible that the algorithms would predict the player’s current team, instead of calculating the predicted result directly, we used the **predict_proba** method to identify the top two most likely targets. If the top target was the player’s current team, then we predicted they would move to the team with the second highest probability. After the predictions were generated, we calculated the F1 score and accuracy using Scikit-learn’s **accuracy_score** and **f1_score**. For the latter, we used the **macro** average which counts the total number of false positives, true positives, and false negatives over each team. The accuracy shown in this paper is the mean of the accuracy over these 100 runs.

## 5 Results

Before presenting the results of this paper, we recall the overarching question “Does a player’s social-professional network influence which team the player transitions to?” As described in the previous section we apply a variety of machine learning techniques with and without social network information as a feature to answer this question. We note that in both sports the number of teams is 30. However, once we have identified a given player as transitioning to a new team we prohibit the player from transitioning to their current team. Hence each transitioning player has 29 possible teams to transition to, and the naïve probability of transitioning to a given team is approximately 3.45%.

We note that each experiment was performed 100 times and the presented accuracy and F1 scores are the mean of these 100 experiments. Complete statistical data tables including 95% confidence intervals (corresponding to p-value equal to 0.05) and confusion matrices are available upon request.

As the Twitter data we collected does not contain dates players started following other players this limits the transitions we can predict to the time after it was collected. Hence, we can only predict transitions that happened in 2020–2021 using Twitter data (see Section 5.1). Later we use this data to infer past transitions that happened during the years 2001–2019 (see Section 5.2). The difference between our prediction and inference accuracy gives us a measure of how player’s social activity shifted after they transitioned from one team to another.

### 5.1 Predictive results

Using www.basketball-reference.com and www.baseball-reference.com, we collected player performance and team fitness data for years 2020–2021. We did not gather new Twitter data, so that all Twitter connections in our data set were made before any team transitions occurred.

As mentioned in our summary section, our ability to predict transitions in the NBA using Twitter data is significantly different from our ability to do the same in MLB. When predicting transitions in the NBA we find that including Twitter data allows for a prediction accuracy of up to 20.3%. Using college data also has a significant effect on the accuracy of our predictions giving us an accuracy of up to 17.4%. (see Table 28 in S1 Appendix). Combining both of these social features slightly increases this probability to a maximum of 20.6%.

In contrast, using player performance and team fitness data has little effect on prediction accuracy. In fact, using this information without social data typically causes the prediction accuracy to drop below the probability of 3.45% of randomly choosing the correct team. This suggests that social data alone is useful in predicting where players will transition to in the NBA (see Table 7 as well as the more complete set of data found in Tables 23-25, 28 in the S1 Appendix).

**Table 7:** Basketball prediction results: The predictions accuracy and F1 score is shown for team transition in the NBA during the 2020 season for players who had Twitter accounts before 2020. Each row indicates the feature(s) used. We note that a “yes” in the social column implies Twitter data was used. The “All Social” row includes both Twitter and College data.

Features	Social Twitter	Accuracy Forest	Accuracy Trees	F1 Forest	F1 Trees
Position	No	0.015	0.014	0.005	0.006
	Yes	0.196	0.193	0.161	0.156
Team	No	0.031	0.028	0.022	0.023
	Yes	0.206	0.104	0.171	0.162
Career Length	No	0.063	0.063	0.035	0.034
	Yes	0.199	.200	0.164	0.161
Performance	No	0.017	0.013	0.011	0.009
	Yes	0.186	0.190	0.154	0.156
Rank	No	0.035	0.028	0.025	0.024
	Yes	0.195	0.210	0.158	0.160
Value	No	0.034	0.032	0.023	0.026
	Yes	0.210	0.207	0.17	0.169
Twitter Only	Yes	0.200	0.203	0.155	0.163
College Only	No	0.174	0.172	0.126	0.124
All Social	Yes	0.200	0.206	1.63	0.163
All Features	No	0.045	0.044	0.032	0.034
	Yes	0.187	0.196	0.157	0.163

In MLB the results are essentially the same if we consider nonsocial data. Using player performance and team fitness to predict MLB transitions during the years 2020–2021 results in probabilities that are similar to those seen in the NBA predictions. The slight difference is that, whereas the NBA probabilities typically drop beneath 3.45% when including nonsocial data, MLB probabilities climb a few percent above this number on average. The major difference, between the NBA and MLB is that prediction accuracy is very low in MLB compared to the NBA when using social data. Using Twitter data in the NBA allows us to achieve accuracy up to 20.3%. In MLB the maximum accuracy we achieve using our algorithms and social data, by itself, is 4.6% only a percent higher than a random guess. The highest predictive accuracy we achieve in MLB is 6.5% when we use only the player’s position. In fact, adding social data often decreases our ability to predict player transitions suggesting that, at least in predicting future transitions, social-professional connections have little to do with a player’s transition from team to team in MLB. (See Table 8 as well as the more complete set of data found in Tables 17–19 in S1 Appendix).

**Table 8:** Baseball prediction results: The prediction accuracy and F1 score is shown for team transition in MLB during 2020 season for players who had Twitter accounts. Each row indicates which feature(s) were used.

Features	Social	Accuracy Forest	Accuracy XGB	F1 Forest	F1 XGB
Position	No	0.055	0.065	0.010	0.010
	Yes	0.046	0.041	0.151	0.176
Team ID	No	0.052	0.056	0.021	0.022
	Yes	0.054	0.038	0.160	0.204
Career Length	No	0.056	0.067	0.025	0.024
	Yes	0.046	0.035	0.151	0.178
Performance	No	0.040	0.040	0.029	0.030
	Yes	0.047	0.037	0.146	0.170
Rank	No	0.059	0.062	0.025	0.024
	Yes	0.050	0.041	0.157	0.178
Value	No	0.056	0.049	0.025	0.026
	Yes	0.046	0.040	0.151	0.173
Social Only	Yes	0.046	0.039	0.155	0.176
All Features	No	0.047	0.041	0.038	0.033
	Yes	0.051	0.039	0.146	0.187

### 5.2 Inferring prior transitions

Since our Twitter data does not contain individual time-stamps indicating when one player starts to follow another, it is not possible to use this data to predict transitions that happened before this data was collected. However, it is possible to use the current state of the Twitter data we can collect to infer which transitions have already happened. That is, we can test to see if Twitter data contains enough information to reconstruct which transitions have already taken place.

#### 5.2.1 Basketball results

A summary of the results inferring prior transitions in the NBA can be found in Table 9, and a more complete summary can be found in Tables 20–22 in S1 Appendix. In Table 9 we see that adding social data improves performance remarkably. Similar to our previous results regarding future predictions nonsocial features had very little impact on accuracy. Performance data alone is about the same as randomly guessing, while using only social data results in a much higher accuracy. In fact, adding social data improves accuracy across all features, and using only social data is worse than using social data with any other feature. Using Twitter data with each nonsocial feature results in a 28–29% accuracy in all cases. As this is higher than our future prediction accuracy this suggests that once players move teams their online activity shifts to indicate the new team they are on. Presumably the players begin to follow other players on their new team.

**Table 9:** **Basketball inference and prediction results: (Top) The inference accuracy and F1 score is shown for team transition in the NBA during the time period 2001–2019 for players who had Twitter accounts**. Each row indicates the feature(s) used. We note that a “yes” in the social column means Twitter data was used. (Bottom) The prediction accuracy and F1 score using the players’ college data to predict team transitions during 2001–2019 is shown.

Features	Social Twitter	Accuracy Forest	Accuracy Trees	F1 Forest	F1 Trees
Position	No	0.040	0.039	0.008	0.008
	Yes	0.294	0.291	0.302	0.302
Team	No	0.042	0.040	0.028	0.028
	Yes	0.290	0.275	0.292	0.273
Career Length	No	0.047	0.050	0.028	0.031
	Yes	0.295	0.296	0.300	0.301
Performance	No	0.037	0.037	0.034	0.033
	Yes	0.287	0.294	0.287	0.298
Rank	No	0.031	0.033	0.022	0.024
	Yes	0.281	0.288	0.286	0.293
Value	No	0.051	0.050	0.046	0.045
	Yes	0.292	0.294	0.296	0.298
Twitter Only	Yes	0.293	0.292	0.298	0.297
All Features	No	0.087	0.085	0.084	0.083
	Yes	0.273	0.282	0.276	0.282
College Only	No	0.084	0.086	0.074	0.076

We also investigated how the use of *college data*, which we consider to be a form of social data, effects the predictiveness of our algorithms. For the years 2001–2019, college data is somewhat advantageous over Twitter data as it allows us to make future predictions regarding transitions rather than inferring them. We find that using college data increased prediction accuracy but not as much as inferring these transitions by using social data.

**Fig 4:**
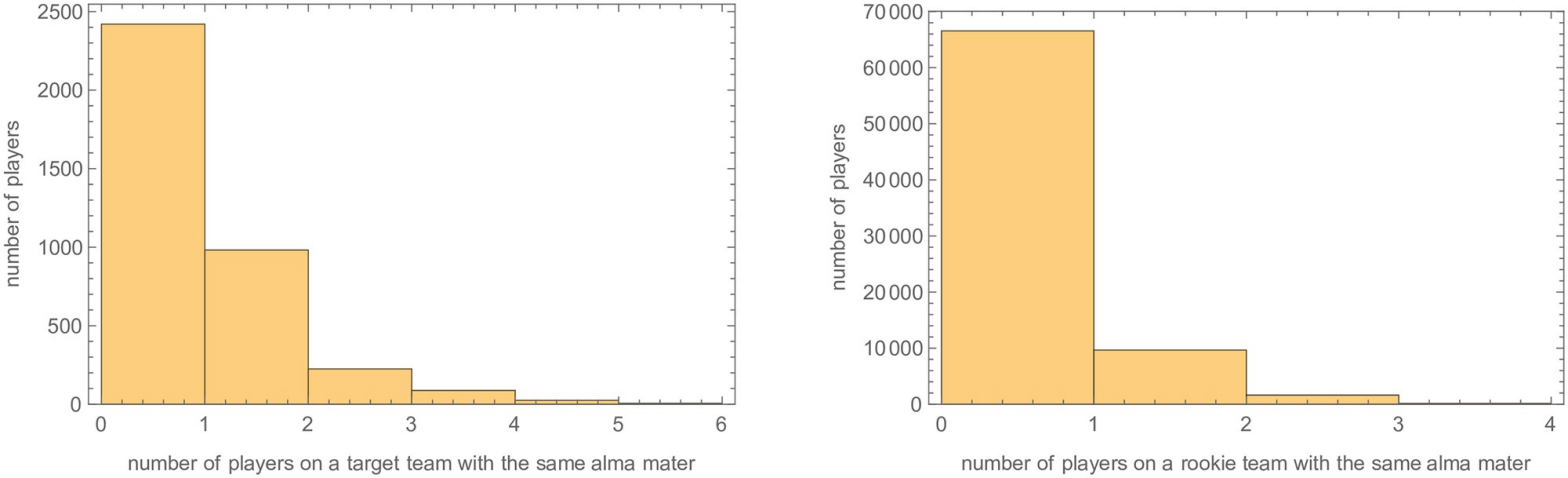
Left: A histogram of the number of players on the target team of a switching player who attended that player’s alma mater. Right: A histogram of the number of players on a rookie’s team who attended the same alma mater.

There are potentially two reasons for this. First, inferring prior transitions may be easier than predicting future transition in general. Second the decrease in accuracy may be due to the fact that even the most frequently attended schools have at most about a dozen active players each year (see Table 29 in S1 Appendix). To give some indication of the prevalence of a fellow alumni on the target team (i.e., the team being transitioned to), for every transitioning player we counted the number of alumni on the target team. As seen in the left panel of Fig 4, in less than half of the transition cases, a fellow alumni is on the team. In the right panel of Fig 4 we considered the slightly different transition problem of whether the prevalence of a fellow alumni influenced what team a rookie player begins on. As can be seen in that histogram, it is likely that alumni connections do not influence initial placement.

Accuracy and F1 score increased using Random Forests, XGB, KNN, and Extra Trees but fluctuated slightly using ADA and Logistic Regression. A complete listing of these scores are included in the Tables 26 and 27 in S1 Appendix.

#### 5.2.2 Baseball results

We summarize the results of our machine learning experiments for inferring prior transitions in the MLB in Table 10. In the table each row indicates which features are used. For instance, in the first row only knowledge of the player’s position is used to predict where the player transitions to. In the second row both the player’s position and the player’s social network, i.e. affinity scores, are used. A more complete summary of the data can be found in Tables 14–16 located in the S1 Appendix. Here, we observe that including social data always has a positive effect, bringing the algorithms’ maximum accuracy up to 17%. This is statistically significant (see the 95% confidence intervals in Table 16 in S1 Appendix). Moreover, each of the nonsocial features yield approximately the same accuracy level, and the combination of all these features does not significantly improve any algorithm’s accuracy. This suggests that these individual features are either in some sense *linearly dependent*, i.e. they are imparting the same information about a particular player, or that the features work against each other in some way. The F1 scores follow the same pattern as the accuracy, with the highly accurate models having the highest F1 scores.

**Table 10:** Baseball inference results: The inference accuracy and F1 score is shown for team transition in MLB during the time period 2002–2019 for players who had Twitter accounts. Each row indicates which feature(s) were used. We note that a “yes” in the social column means Twitter data was used.

Features	Social	Accuracy Forest	Accuracy XGB	F1 Forest	F1 XGB
Position	No	0.044	0.044	0.01	0.01
	Yes	0.171	0.187	0.151	0.176
Team ID	No	0.032	0.031	0.021	0.022
	Yes	0.179	0.218	0.160	0.204
Career Length	No	0.051	0.050	0.025	0.024
	Yes	0.172	0.189	0.151	0.178
Performance	No	0.032	0.036	0.029	0.030
	Yes	0.166	0.181	0.146	0.170
Rank	No	0.039	0.039	0.025	0.024
	Yes	0.177	0.190	0.157	0.178
Value	No	0.041	0.043	0.025	0.026
	Yes	0.170	0.184	0.151	0.173
Social Only	Yes	0.174	0.187	0.155	0.176
All Features	No	0.041	0.040	0.038	0.033
	Yes	0.169	0.204	0.146	0.187

### 5.3 A temporal comparison

Using Twitter data to determine transitions in the NBA and MLB has at least two drawbacks. The first, already mentioned, is that this data is not time-stamped. The second is that Twitter was not founded until 2006, and although players from the early years of our study have joined Twitter a much lower percentage of these players have accounts and thus our proxy social-professional network is less complete for those years (see Fig 1).

With this in mind we considered one additional test of the efficacy of using Twitter data by comparing the accuracy of our machine learning algorithms for the *earlier years* (2001–2010) and the *later years* (2011–2019). The results are shown in Tables 11 and 12 for MLB and the NBA, respectively. In every case considered in these tables, if social data is used the algorithm’s accuracy is significantly higher in the later time period when Twitter usage is higher than in the earlier time period (see Fig 1). For baseball, using Twitter data alone increases accuracy from around 10% to 20% as the average Twitter usage climbs from 16.4% to 45.3% during 2002–2009 and 2010–2018, respectively. For basketball, accuracy increases from about 21% to 31% as the average Twitter usage climbs from 28.7% to 64.3% during 2001–2009 and 2010–2019, respectively. This suggests that the more complete our information is on the social interactions of players the better we can infer and possibly predict their transitions.

**Table 11:** **Basketball inference and prediction accuracy: (Top) The inference accuracy using Random Forest for team transition in NBA during the time periods 2002–2010, 2011–2019, and 2002–2019 is shown**. (Bottom) The prediction accuracy using the players’ college data to predict team transitions during 2001–2019 is shown.

Features	Social	2001–2010 Accuracy	2011–2019 Accuracy	2002–2019 Accuracy
Position	No	0.041	0.045	0.04
	Yes	0.222	0.327	0.294
Team	No	0.059	0.048	0.042
	Yes	0.222	0.324	0.29
Career Length	No	0.042	0.050	0.047
	Yes	0.218	0.328	0.295
Performance	No	0.033	0.044	0.037
	Yes	0.202	0.309	0.287
Rank	No	0.039	0.044	0.031
	Yes	0.202	0.319	0.281
Value	No	0.045	0.052	0.051
	Yes	0.213	0.319	0.292
Twitter Only	Yes	0.215	0.325	0.293
All Features	No	.108	0.081	0.087
	Yes	0.204	0.308	0.276
College Only	No	0.098	0.092	0.084

**Table 12:** Baseball inference accuracy: The inference accuracy using XGBoost for team transition in MLB during the time periods 2002–2010, 2011–2019, and 2002–2019 is shown.

Features	Social	2002–2010 Accuracy	2011–2019 Accuracy	2002–2019 Accuracy
Position	No	0.043	0.05	0.044
	Yes	0.106	0.189	0.187
Team ID	No	0.041	0.041	0.031
	Yes	0.098	0.224	0.218
Career Length	No	0.037	0.047	0.050
	Yes	0.098	0.193	0.189
Performance	No	0.035	0.035	0.036
	Yes	0.095	0.182	0.181
Rank	No	0.021	0.04	0.039
	Yes	0.098	0.194	0.190
Value	No	0.045	0.046	0.043
	Yes	0.103	0.19	0.184
Social Only	Yes	0.107	0.191	0.187
All Features	No	0.047	0.04	0.04
	Yes	0.095	0.206	0.204

## 6 Network analysis of the Twitter MLB and NBA data sets

In this section we investigate the properties of both the MLB Twitter and NBA Twitter networks described in Section 3. We first consider the basic statistical properties of these networks and then compare their degree, eigenvector, closeness, and betweenness centralities.

The basic network statistics we consider are the network’s total number of nodes *n*, number of directed edges *m*, mean degree *c*, fraction of nodes in the largest strongly connected component *S*, mean distance between connected node pairs *ℓ*, clustering coefficient *C*, reciprocity *r*, and the degree assortativity *a*. The *mean degree* of the network is *c* = *m*/*n*. A *strongly connected component* of a network is a maximal set of nodes such that it is possible to reach any node from any other node. If the largest of these components has *n*_*max*_ nodes then *S* = *n*_*max*_/*n*. The *distance* *d*_*ij*_ from node *i* to node *j* is the length of the shortest path from node *i* to node *j* through the network. If such a path exists we say node *i* is *connected* to node *j*. The average *ℓ* = 〈*d*_*ij*_〉 over all connected nodes is the network’s *mean distance* between connected nodes. The *clustering coefficient* *C* is, roughly speaking, the fraction of triangles in the network versus “potential triangles” or paths of length 2. The network *reciprocity* is the percentage of edges that are reciprocated or, for our networks, how often a player follows someone that follows them. Last, if the tendency is for players that follow many players to follow those that also follow many players then the network is said to be *assortative* where 0 < *a* ≤ 1. Otherwise, the network is *disassortative* with −1 ≤ *a* < 0. (For a more detailed description of these network quantities see [32]).

In Table 13 these statistics are shown for both networks. Although the number of nodes and edges in these networks are, relatively speaking, quite different each of the other statistics in the table are very similar. In fact, it is striking how similar some of these statistics are. This suggests that these two networks have very similar structures which in turn suggests that the reason we have better predictions for the NBA versus the MLB is not due to specific structural features of these networks.

**Table 13:** Basic statistics for the MLB and NBA Twitter networks using mathematica.

Network	*n*	*m*	*c*	*S*	*ℓ*	*C*	*r*	*a*
MLB	1364	76977	56.43	0.931	2.07	0.189	0.605	-0.043
NBA	1003	58750	58.57	0.971	2.15	0.190	0.614	-0.023

Properties measured are: total number of nodes *n*, number of directed edges *m*; mean degree *c*; fraction of nodes in the largest strongly connected component *S*; mean distance between connected node pairs *ℓ*; clustering coefficient *C*; reciprocity *r*, and the degree assortativity *a*.

**Fig 5:**
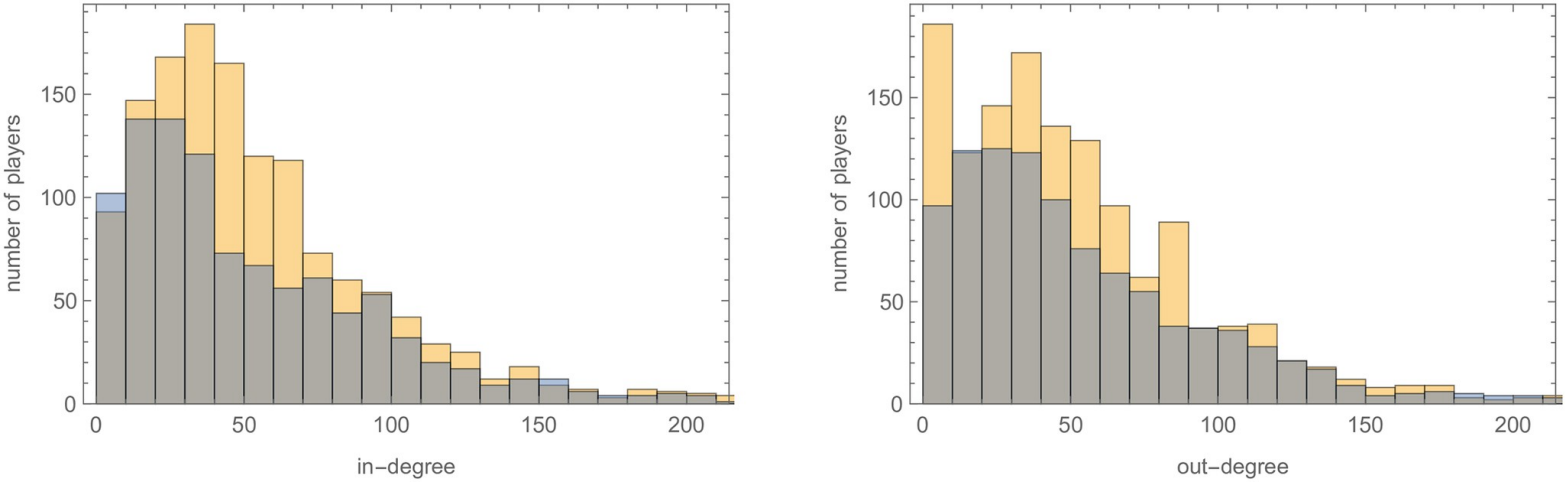
A histogram of the in-degree and out-degree centrality for the baseball and basketball-Twitter networks are shown left and right, respectively. Baseball is shown in orange and basketball is shown in blue in each histogram.

**Fig 6:**
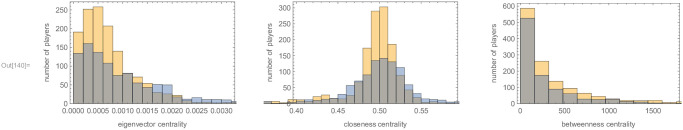
Histograms of the eigenvector centrality (EC), closeness centrality (CC), and betweenness centrality (BC) for the baseball and basketball-Twitter networks shown left, center, and right, respectively. Baseball is shown in orange and basketball is shown in blue in each histogram.

To give more evidence to the notion that the baseball Twitter and basketball Twitter networks have a similar structure, we note that the distribution of the networks’ degrees (Fig 2), in-degrees, and out-degrees (Fig 5) have very similar shapes and that the same holds for the networks’ eigenvector, closeness, and betweenness centralities (Fig 6). Here an individual’s *in-degree* is the number of Twitter followers they have while *out-degree* is the number of player’s they follow. An individual’s *eigenvector centrality* is high if they are followed by players that collectively have a high centrality. To have high *closeness centrality* a player’s mean distance to all other players in the network should be small. To have high *betweenness centrality* the player should be on many of the shortest paths between other pairs of players.

## 7 Limitations

In this paper we have considered using social networks to predict and infer player transitions in both professional basketball and baseball. Naturally, limitations in the data available, differences between the two sports, and our desire to have a similar model for both sports put constraints on our analysis. In this section we detail a number of these constraints and their consequences.

Perhaps the largest constraint comes from the use of Twitter data as a proxy for our two social networks. Although Twitter allows us to create an approximation of the players’ social-professional network, this data is not time stamped. Consequently, it is not possible to determine whether a social connection existed before or after a transition was made between teams.

It is possible to avoid this issue by gathering data at regular intervals or even at the end of a “typical” year. This, now time-stamped, data could then be used to predict transitions in the following year without the ambiguity of knowing whether the social connection preceded the transition or not. One drawback to this strategy is that we may need to wait for a typical year. The 2020–2021 transitions are potentially quite distinct from any of the previous years due to the Covid-19 pandemic’s effect on the MLB and NBA. However, our results suggest that using time-stamped social data together with the methods introduced in this paper could further extend our understanding of the influence of social interactions on group transitions in general social-professional networks.

Aside from the temporal limitations of our data, one limitation of our analysis is that we do not differentiate between free agent movement and trades between teams. The primary reason for this is the difference in contracts between the two sports, both in terms of obligated contract length and the way that free agency works. In baseball, players are contractually obligated to their teams for longer periods of time, and players remain contractually obligated to that team even if their contract to play is not renewed. In basketball, players can be considered as either unrestricted free agents or restricted free agents. In order to take into account the differences between the two leagues we would need to treat them separately, track trade combinations, i.e., these two players are traded for those three players, etc. and track when players resign with a team due to their restricted status.

Another limitation is that, although the financial health of the teams is considered, we did not consider the salaries of players who transitioned between teams. In baseball, it is possible for a financially healthy team to acquire a very good player by offering a high salary despite having other highly paid players, but this is much less likely in basketball where salary caps are strictly enforced. In our analysis we ignore this difference. In a more detailed analysis it might be possible to track the salary cap space, along with player’s current contract numbers, or projected worth to determine if it is even possible for a player to join a given team. For this to work well, we would have to track the order in which transitions occur as teams release players to make salary cap room for a star player.

## 8 Conclusion

In this paper we consider the question “Do social connections influence professional group transition?” in the context of both Major League Baseball and the National Basketball Association. Specifically, we analyze to what extent social connections can help predict how players change teams. We find that the addition of social data significantly improve the accuracy of our results. In particular we compare which of the following types of data *player performance*, *team fitness*, and *social data* are more predictive in the context of machine learning. We find that the addition of player performance and team fitness data can both slightly improve and slightly decrease the performance of our algorithms but overall has little effect on prediction and inference accuracy. In contrast, the use of social data significantly improves our ability to predict future transitions in the NBA bringing our accuracy up to 20%. In MLB the results are quite different as including social data does little to improve accuracy and in many cases actually degrades our accuracy.

For inferring past transitions the use of social data does improve our predictions for both the NBA and MLB. In fact, our highest accuracies were obtained in this manner. This suggests that once players shift teams their online activities shift in a way that indicates this transition. The difference between prediction and inference accuracy gives us a sense of how large this shift is.

The fact that performance and team data did little to change our scores is, to us, a bit surprising. There may be several reasons for this lack of improvement. In separate experiments, we discovered that performative data does influence the likelihood of a player not returning to play the following year. That is, performance data seems to be better suited to answer “if” a player will leave a team rather than “where” the player will go. This is important in the sense that the number of players leaving the MLB and NBA is nearly equal to the number of players transitioning most years.

We also note that the social networks under consideration are strikingly similar, hence the differences in prediction accuracy between baseball and basketball are likely not due to network structure. We conjecture that the differences in accuracy with the inclusion of social data between baseball and basketball may, in fact, be partially due to the percentage of players for which we have social data. As further evidence we compare the accuracy of our machine learning algorithms on the early years of the data versus the later years of the data. Both baseball and basketball show an increase in the percentage of players with social information and also an increase in the accuracy of the algorithm in the later years. Again, we temper these results with a reminder that these social networks were created from unstamped Twitter data and consequently, it was not possible to determine whether a social connection existed before or after a transition was made between teams.

To counter this limitation, we also consider college attendance as a proxy for social-professional network in the NBA. We find that this can also increase the prediction accuracy of our chosen algorithms. We were able to obtain college information for a larger percentage of our basketball players, and although the accuracy of the results did not improve as much as when we used Twitter data it did improve more than using any other non-social data.

As mentioned, empirical data from early experiments show that performance data is a good indicator of retirement. Future work includes quantifying these results, and also investigating if a strong social network helps to delay retirement. An interesting question to investigate is whether the inclusion of external social networks, for example between college and professional level coaches, would impact the results. Finally, we hope to extend our results to other types of professional groups including groups that make up academic networks and industry networks to see if the impact of social networks is the same.

## Supporting information

S1 Appendix Supplementary material for using social networks to improve team transition prediction in professional sports. The supplementary material includes informative data that extends the data presented in the main body of the work. It includes a complete summary information for all of the machine learning algorithms utilized, and all of the combinations of features. It also includes the tables of the most socially active players in both baseball and basketball for all of the centralities we consider.
(PDF)
