# Supplementary material for: Using social networks to improve team transition prediction in professional sports
Source: PLoS One. 2022 Jun 24;17(6):e0268619. doi: 10.1371/journal.pone.0268619 (PMC9231699; doi:10.1371/journal.pone.0268619)
Supplement: S1 Appendix — The supplementary material includes informative data that extends the data presented in the main body of the work. It includes a complete summary information for all of the machine learning algorithms utilized, and all of the combinations of features. It also includes the tables of the most socially active players in both baseball and basketball for all of the centralities we consider. (PDF) [file pone.0268619.s001.pdf]

Supplementary material for using social networks to improve team transition prediction in professional sports

Emily J. Evans[1], Rebecca Jones[1], Joseph Leung[1], Benjamin Z. Webb[1]

1 Department of Mathematics, Brigham Young University, Provo, Utah 84602, USA

* bwebb@mathematics.byu.edu

The supplementary material includes informative data that extends the data presented in the main body of the work. It includes a complete summary information for all of the machine learning algorithms utilized, and all of the combinations of features. It also includes the tables of the most socially active players in both baseball and basketball for all of the centralities we consider.

Features	Social Data	ADA	Logistic Regression	Random Forest	XGBoost	KNN	Extra Trees
Positions	N	4.2%	4.2%	4.4%	4.4%	3.9%	4.5%
Only	Y	17.1%	15.4%	17.1%	18.7%	7.7%	13.1%
Team	N	3.8%	3.0%	3.2%	3.1%	3.7%	3.3%
Only	Y	17.4%	16.7%	17.9%	21.8%	5.5%	13.7%
Career	N	5.1%	4.7%	5.1%	5.0%	4.2%	5.3%
Length Only	Y	16.7%	16.7%	17.2%	18.9%	9.0%	14.2%
Performance	N	4.0%	3.7%	3.2%	3.6%	3.3%	3.2%
Only	Y	16.7%	15.1%	16.6%	18.1%	8.2%	13.4%
Rank	N	4.4%	4.1%	3.9%	3.9%	4.0%	3.7%
Only	Y	17.2%	16.0%	17.7%	19.0%	9.0%	14.1%
Value	N	3.8%	3.5%	4.1%	4.3%	4.0%	4.0%
Only	Y	17.1%	16.1%	17.0%	18.4%	8.2%	14.0%
Social Only	Y	17.4%	16.0%	17.4%	18.7%	9.0%	14.0%
All data	N	4.7%	3.9%	4.1%	4.0%	3.8%	4.0%
	Y	15.8%	15.8%	16.9%	20.4%	4.7%	13.0%

S1 Table 14. Summary of Algorithm Accuracy for Baseball data from 2001-2019. Using non-social features, the accuracy was typically slightly better than guessing, 3.44%. Using social data, accuracy increased in every case and was 15-21% using Logistic Regression, Random Forest, and XGBoost.

Features	Social Data	ADA	Logistic Regression	Random Forest	XGBoost	KNN	Extra Trees
Positions	N	0.01	0.009	0.01	0.01	0.008	0.01
Only	Y	0.157	0.146	0.151	0.176	0.070	0.117
Team	N	0.008	0.020	0.021	0.022	0.022	0.020
Only	Y	0.160	0.158	0.160	0.204	0.049	0.121
Career	N	0.016	0.008	0.025	0.024	0.021	0.026
Length Only	Y	0.150	0.155	0.151	0.178	0.080	0.125
Performance	N	0.012	0.017	0.029	0.030	0.030	0.028
Only	Y	0.152	0.141	0.146	0.170	0.073	0.118
Rank	N	0.012	0.007	0.025	0.024	0.025	0.023
Only	Y	0.159	0.151	0.157	0.178	0.082	0.125
Value	N	0.010	0.005	0.025	0.026	0.025	0.024
Only	Y	0.158	0.152	0.151	0.173	0.073	0.124
Social Only	Y	0.158	0.152	0.155	0.176	0.081	0.124
All data	N	0.018	0.033	0.038	0.033	0.036	0.037
	Y	0.14	0.149	0.146	0.187	0.042	0.113

S1 Table 15. Summary of Algorithm F1 for Baseball data for 2002-2019. We observe that the highest F1 score was obtained using the same model that achieved the highest accuracy.

Features	Social Data	ADA	Logistic Regression	Random Forest	XGBoost	KNN	Extra Trees
Positions	N	(.03, .055)	(.023, .057)	(.028, .06)	(.029, .059)	(.021, .056)	(.029, .06)
Only	Y	(.142, .201)	(.122, .182)	(.147, .198)	(.158, .221)	(.052, .10)	(.101, .158)
Team	N	(.024, .051)	(.019, .042)	(.021, .045)	(.021, .043)	(.023, .052)	(.021, .046)
Only	Y	(.137, .216)	(.137, .203)	(.152, .206)	(.187, .245)	(.035, .071)	(.112, .167)
Career	N	(.033, .07)	(.029, .064)	(.037, .065)	(.031, .066)	(.027, .062)	(.035, .068)
Length Only	Y	(.136, .206)	(.146, .195)	(.145, .198)	(.161, .218)	(.066, .112)	(.114, .172)
Performance	N	(.021, .054)	(.023, .054)	(.019, .043)	(.023, .052)	(.022, .05)	(.018, .046)
Only	Y	(.139, .203)	(.125, .176)	(.142, .193)	(.147, .21)	(.062, .104)	(.108, .158)
Rank	N	(.027, .06)	(.024, .06)	(.024, .056)	(.027, .052)	(.026, .06)	(.024, .05)
Only	Y	(.139, .211)	(.133, .189)	(.149, .203)	(.165, .214)	(.069, .114)	(.116, .168)
Value	N	(.025, .054)	(.021, .048)	(.026, .054)	(.024, .059)	(.026, .056)	(.028, .055)
Only	Y	(.131, .212)	(.133, .184)	(.14, .199)	(.16, .21)	(.062, .102)	(.118, .165)
Social Only	Y	(.133, .217)	(.131, .186)	(.153, .203)	(.151, .217)	(.07, .113)	(.116, .172)
All data	N	(.031, .06)	(.024, .058)	(.026, .056)	(.023, .057)	(.025, .052)	(.026, .058)
	Y	(.126, .191)	(.129, .19)	(.143, .195)	(.177, .236)	(.033, .064)	(.109, .161)

S1 Table 16. Summary of 95% confidence intervals for Baseball data for 2002-2019 over 100 runs.

Features	Social Data	ADA	Logistic Regression	Random Forest	XGBoost	KNN	Extra Trees
Positions	N	5.1%	4.7%	5.5%	6.5%	3.9%	6.2%
Only	Y	4.4%	5.0%	4.6%	4.1%	3.8%	4.4%
Team	N	5.2%	5.3%	5.2%	5.6%	5.1%	5.7%
Only	Y	5.2%	5.2%	5.4%	3.8%	4.9%	4.7%
Career	N	6.4%	3.5%	5.6%	6.7%	5.2%	5.1%
Length Only	Y	4.6%	4.6%	4.6%	3.5%	3.6%	4.3%
Performance	N	3.5%	2.9%	4.0%	4.0%	5.0%	4.0%
Only	Y	4.4%	4.7%	4.7%	3.7%	3.1%	4.4%
Rank	N	5.0%	3.1%	5.9%	6.2%	5.1%	5.9%
Only	Y	4.5%	3.9%	5.0%	4.1%	4.0%	4.7%
Value	N	4.5%	2.8%	5.6%	4.9%	5.2%	5.3%
Only	Y	5.2%	4.7%	4.6%	4.0%	3.2%	4.9%
Social Only	Y	4.9%	5.0%	4.6%	3.9%	3.6%	5.1%
All data	N	5.5%	4.2%	4.7%	4.1%	4.4%	5.4%
	Y	5.2%	4.0%	5.1%	3.9%	5.4%	4.4%

S1 Table 17. Summary of Algorithm Accuracy for Baseball data from 2020.

Features	Social Data	ADA	Logistic Regression	Random Forest	XGBoost	KNN	Extra Trees
Positions	N	0.015	0.015	0.017	0.019	0.012	0.019
Only	Y	0.014	0.029	0.02	0.023	0.021	0.021
Team	N	0.009	0.032	0.031	0.007	0.029	0.032
Only	Y	0.014	0.031	0.025	0.019	0.024	0.026
Career	N	0.017	0.006	0.024	0.030	0.023	0.022
Length Only	Y	0.014	0.026	0.020	0.017	0.023	0.021
Performance	N	0.014	0.013	0.026	0.024	0.026	0.027
Only	Y	0.013	0.028	0.022	0.020	0.018	0.022
Rank	N	0.016	0.009	0.037	0.035	0.028	0.035
Only	Y	0.013	0.024	0.021	0.023	0.024	0.023
Value	N	0.009	0.006	0.033	0.030	0.029	0.030
Only	Y	0.018	0.028	0.02	0.022	0.019	0.126
Social Only	Y	0.016	0.028	0.22	0.021	0.021	0.026
All data	N	0.019	0.024	0.029	0.025	0.026	0.034
	Y	0.015	0.024	0.026	0.023	0.030	0.023

S1 Table 18. Summary of Algorithm F1 for Baseball data for 2020.

Features	Social Data	ADA	Logistic Regression	Random Forest	XGBoost	KNN	Extra Trees
Positions	N	(.0, .118)	(.0, .088)	(.0, .118)	(.0, .0118)	(.0, .118)	(.0, .118)
Only	Y	(.0, .088)	(.0, .118)	(.0, .104)	(.0, .088)	(.0, .088)	(.0, .133)
Team	N	(.0, .118)	(.0, .118)	(.0, .118)	(.0, .118)	(.0, .133)	(.0, .118)
Only	Y	(.0, .133)	(.0, .118)	(.0, .133)	(.0, .088)	(.0, .088)	(.0, .118)
Career	N	(.0, .147)	(.0, .118)	(.0, .147)	(.0, .147)	(.0, .118)	(.0, .118)
Length Only	Y	(.0, .118)	(.0, .118)	(.0, .104)	(.0, .088)	(.0, .104)	(.0, .104)
Performance	N	(.0, .104)	(.0, .088)	(.0, .088)	(.0, .104)	(.0, .118)	(.0, .088)
Only	Y	(.0, .133)	(.0, .118)	(.0, .118)	(.0, .104)	(.0, .088)	(.0, .118)
Rank	N	(.0, .118)	(.0, .088)	(.0, .133)	(.0, .133)	(.0, .088)	(.0, .118)
Only	Y	(.0, .133)	(.0, .088)	(.0, .118)	(.0, .118)	(.0, .118)	(.0 .118)
Value	N	(.0, .118)	(.0, .088)	(.0, .118)	(.0, .088)	(.0, .118)	(.0, .118)
Only	Y	(.0, .118)	(.0, .118)	(.0, .118)	(.0, .088)	(.0, .088)	(.0, .118)
Social Only	Y	(.0, .118)	(.0, .147)	(.0, .118)	(.0, .104)	(.0, .104)	(.0, .118)
All data	N	(.0, .118)	(.0, .104)	(.0, .118)	(.0, .118)	(.0, .118)	(.0, .118)
	Y	(.0, .118)	(.0, .118)	(.0, .118)	(.0, .088)	(.0, .133)	(.0, .118)

S1 Table 19. Summary of 95% confidence intervals for Baseball data for 2020 over 100 runs.

Data Used	Twitter	College	ADA	Logistic Regression	Random Forest	XGBoost	KNN	Extra Trees
Positions Only	N	N	4.1%	3.4%	4.0%	4.1%	3.4%	3.9%
	N	Y	4.2%	3.6%	10.9%	8.1%	10.7%	10.9%
	Y	N	9.3%	19.6%	29.4%	23.7%	23.0%	29.1%
	Y	Y	9.4%	19.4%	**29.9%**	23.7%	23.4%	29.8%
Team Only	N	N	3.9%	4.0%	4.2%	3.9%	3.6%	4.0%
	N	Y	3.8%	3.9%	9.0%	6.0%	8.5%	8.7%
	Y	N	9.9%	18.1%	29%	21.9%	10.2%	27.5%
	Y	Y	9.9%	17.8%	29.6%	22.2%	10.8%	28.3%
Career Length Only	N	N	4.5%	4.4%	4.7%	4.7%	4.0%	5.0%
	N	Y	4.5%	4.3%	13.9%	8.8%	13.8%	14.3%
	Y	N	9.3%	20.3%	29.5%	23.8%	24.3%	29.6%
	Y	Y	9.0%	20.2%	29.9%	23.9%	24.7%	29.9%
Performance Only	N	N	3.6%	3.4%	3.7%	3.7%	3.9%	3.7
	N	Y	4.1%	3.5%	4.9%	4.9%	4.1%	6.2%
	Y	N	9.0 %	19.9%	28.7%	20.7%	21.9%	29.4%
	Y	Y	9.0%	19.9%	28.7%	20.7%	21.9%	29.4%
Rank Only	N	N	3.9%	3.0%	3.1%	3.3%	3.5%	3.3%
	N	Y	4.0%	3.3%	4.3%	5.7%	4.2%	4.5%
	Y	N	9.9%	19.8%	28.1%	21.9%	21.9%	28.8%
	Y	Y	10.0%	19.6%	28.1%	22.1%	22.7%	28.9%
Valuation Only	N	N	4.1%	3.5%	5.1%	4.2%	4.6%	5.0%
	N	Y	3.9%	3.4%	8.5%	6.3%	7.8%	9.4%
	Y	N	9.3%	19.9%	29.2%	22.3%	23.5%	29.4%
	Y	Y	9.2%	19.9%	29.4%	22.4%	24.0%	29.9%
Twitter	Y	N	9.2%	20.2%	29.3%	23.5%	23.5%	29.2%
College	N	Y	4.3%	2.9%	8.4%	7.4%	7.2%	8.6%
All Social	Y	Y	9.5%	19.9%	29.6%	23.5%	24.2%	29.7%
All Data	N	N	4.0%	4.3%	8.7%	6.2%	7.0%	8.5%
	N	Y	4.0%	4.2%	8.7%	6.2%	7.1%	8.4%
	Y	N	9.0%	17.9%	27.3%	19.6%	11.1%	28.2%
	Y	Y	9.2%	17.5%	27.6%	20.3%	11.3%	28.5%

S1 Table 20. Summary of algorithm accuracy for basketball data for 2001-2019.

Data Used	Twitter	College	ADA	Logistic Regression	Random Forest	XGBoost	KNN	Extra Trees
Positions Only	N	N	0.008	0.011	0.008	0.008	0.001	0.008
	N	Y	0.012	0.017	0.103	0.072	0.103	0.104
	Y	N	0.081	0.189	0.302	0.241	0.232	0.302
	Y	Y	0.082	0.189	0.302	0.241	0.232	0.302
Team Only	N	N	0.008	0.029	0.028	0.027	0.023	0.027
	N	Y	0.009	0.030	0.087	0.056	0.083	0.084
	Y	N	0.084	0.173	0.292	0.221	0.1	0.273
	Y	Y	0.085	0.169	0.296	0.224	0.105	0.284
Career Length Only	N	N	0.012	0.008	0.028	0.027	0.025	0.031
	N	Y	0.016	0.015	0.134	0.082	0.134	0.138
	Y	N	0.079	0.198	0.300	0.245	0.241	0.301
	Y	Y	0.077	0.195	**0.305**	0.243	0.247	0.305
Performance Only	N	N	0.009	0.018	0.034	0.033	0.036	0.033
	N	Y	0.011	0.020	0.044	0.044	0.037	0.057
	Y	N	0.081	0.190	0.287	0.209	0.212	0.298
	Y	Y	0.077	0.192	0.292	0.21	0.218	0.299
Rank Only	N	N	0.012	0.005	0.022	0.024	0.024	0.024
	N	Y	0.015	0.011	0.042	0.054	0.041	0.044
	Y	N	0.088	0.195	0.286	0.224	0.218	0.293
	Y	Y	0.088	0.192	0.285	0.226	0.226	0.294
Valuation Only	N	N	0.010	0.006	0.046	0.035	0.040	0.045
	N	Y	0.012	0.011	0.082	0.058	0.075	0.091
	Y	N	0.079	0.193	0.296	0.228	0.232	0.298
	Y	Y	0.078	0.193	0.298	0.229	0.239	0.305
Twitter	Y	N	0.082	0.194	0.298	0.242	0.233	0.297
College	N	Y	0.010	0.005	0.074	0.062	0.066	0.076
All Social	Y	Y	0.083	0.192	0.299	0.239	0.241	0.301
All Data	N	N	0.017	0.038	0.084	0.060	0.068	0.083
	N	Y	0.016	0.038	0.083	0.059	0.069	0.082
	Y	N	0.075	0.172	0.276	0.199	0.109	0.282
	Y	Y	0.077	0.168	0.279	0.205	0.111	0.285

S1 Table 21. Summary of algorithm F1 score for basketball data from 2001-2019. The model with the greatest accuracy also had the highest F1 score.

Data Used	Twitter	College	ADA	Logistic Regression	Random Forest	XGBoost	KNN	Extra Trees
Positions Only	N	N	(.028, .058)	(.021, .051)	(.027, .054)	(.026, .053)	(.019, .048)	(.026, .053)
	N	Y	(.026, .061)	(.023, .049)	(.088, .129)	(.061, .099)	(.089, .131)	(.088, .129)
	Y	N	(.068, .124)	(.169, .224)	(.265, .332)	(.215, .261)	(.204, .258)	(.256, .315)
	Y	Y	(.007, .118)	(.168, .221)	(.269, .330)	(.21, .266)	(.203, .266)	(.267, .33)
Team Only	N	N	(.026, .053)	(.028, .053)	(.03, .057)	(.028, .052)	(.025, .048)	(.027, .052)
	N	Y	(.026, .052)	(.026, .054)	(.069, .109)	(.044, .075)	(.068, .107)	(.068, .106)
	Y	N	(.067, .125)	(.155, .216)	(.262, .321)	(.189, .242)	(.081, .126)	(.242, .302)
	Y	Y	(.065, .132)	(.153, .20)	(.269, .324)	(.187, .253)	(.09, .129)	(.251, .314)
Career Length Only	N	N	(.03, .059)	(.027, .057)	(.033, .059)	(.033, .061)	(.025, .058)	(.037, .061)
	N	Y	(.031, .059)	(.028, .058)	(.115, .168)	(.066, .109)	(.113, .16)	(.119, .167)
	Y	N	(.071, .121)	(.178, .229)	(.262, .328)	(.205, .269)	(.215, .266)	(.265, .336)
	Y	Y	(.061, .118)	(.175, .232)	(.268, .329)	(.21, .271)	(.218, .277)	(.27, .33)
Performance Only	N	N	(.023, .051)	(.021, .047)	(.025, .053)	(.025, .049)	(.029, .052)	(.023, .051)
	N	Y	(.026, .057)	(.023, .049)	(.031, .067)	(.039, .061)	(.027, .053)	(.041, .084)
	Y	N	(.064, .128)	(.164, .226)	(.248, .313)	(.177, .233)	(.182, .24)	(.26, .332)
	Y	Y	(.064, .117)	(.162, .225)	(.258, .32)	(.179, .234)	(.189, .247)	(.263, .332)
Rank Only	N	N	(.026, .054)	(.015, .044)	(.02, .041)	(.023, .0444)	(.024, .052)	(.02, .047)
	N	Y	(.027, .055)	(.02, .047)	(.029, .054)	(.04, .074)	(.028, .061)	(.032, .061)
	Y	N	(.074, .125)	(.165, .224)	(.252, .31)	(.196, .246)	(.192, .245)	(.259, .312)
	Y	Y	(.068, .133)	(.172, .22)	(.245, .313)	(.184, .251)	(.197, .256)	(.261, .318)
Valuation Only	N	N	(.028, .057)	(.023, .048)	(.038, .063)	(.028, .054)	(.033, .06)	(.037, .064)
	N	Y	(.027, .054)	(.024, .046)	(.069, .102)	(.041, .083)	(.06, .096)	(.074, .111)
	Y	N	(.066, .115)	(.173, .227)	(.264, .315)	(.196, .249)	(.201, .264)	(.263, .325)
	Y	Y	(.067, .122)	(.176, .226)	(.267, .33)	(.196, .254)	(.215, .269)	(.272, .33)
Twitter	Y	N	(.071, .118)	(.171, .229)	(.253, .323)	(.209, .261)	(.204, .266)	(.265, .323)
College	N	Y	(.025, .057)	(.017, .041)	(.067, .104)	(.055, .096)	(.053, .089)	(.07, .104)
All Social	Y	Y	(.072, .126)	(.167, .226)	(.226, .326)	(.203, .263)	(.213, .272)	(.267, .329)
All Data	N	N	(.025, .056)	(.03, .058)	(.066, .109)	(.042, .079)	(.052, .085)	(.068, .109)
	N	Y	(.025, .057)	(.028, .06)	(.065, .105)	(.046, .077)	(.05, .089)	(.066, .106)
	Y	N	(.062, .116)	(.153, .205)	(.243, .304)	(.172, .225)	(.093, .131)	(.256, .307)
	Y	Y	(.06, .12)	(.148, .202)	(.246, .31)	(.178, .232)	(.089, .133)	(.248, .321)

S1 Table 22. Summary of 95% confidence intervals for the basketball data from 2001-2019. Each algorithm was run 100 times.

Data Used	Twitter	College	ADA	Logistic Regression	Random Forest	XGBoost	KNN	Extra Trees
Positions Only	N	N	2.5%	1.9%	1.4%	2.1%	2.7%	1.7%
	N	Y	4.7%	2.8%	14.8%	14.4%	16.2%	14.6%
	Y	N	5.6%	16.3%	19.9%	18.8%	16.5%	19.6%
	Y	Y	5.0%	18.2%	20.5%	19.8%	17.2%	20.1%
Team Only	N	N	3.5%	3.6%	3.9%	2.8%	3.1%	3.0%
	N	Y	4.4%	3.6%	4.2%	15.2%	4.0%	4.8%
	Y	N	4.6%	9.9%	20.8%	19.5%	9.5%	20.7%
	Y	Y	4.5%	10.0%	20.7%	20.4%	9.1%	20.2%
Career Length Only	N	N	3.7%	3.3%	7.1%	4.4%	5.9%	6.0%
	N	Y	4.6%	4.6%	21.0%	18.4%	20.5%	20.5%
	Y	N	4.7%	17.1%	19.6%	20.5%	20.3%	20.4%
	Y	Y	5.2%	16.7%	21.2%	20.0%	20.5%	20.6%
Performance Only	N	N	2.4%	2.3%	2.0%	3.0%	0.9%	1.1%
	N	Y	3.7%	3.9%	6.4%	6.8%	3.4%	10.5%
	Y	N	4.8%	15.7%	19.1%	15.7%	19.3%	20.6%
	Y	Y	4.5%	17.2%	19.0%	18.2%	20.5%	20.9%
Rank Only	N	N	3.0%	1.8%	3.6%	2.8%	3.0%	3.0%
	N	Y	4.6%	2.9%	4.8%	9.1%	3.7%	6.3%
	Y	N	5.0%	16.3%	21.4%	18.4%	20.7%	20.7%
	Y	Y	4.8%	17.0%	20.8%	18.6%	20.2%	20.5%
Valuation Only	N	N	3.4%	2.2%	3.3%	3.8%	3.0%	2.9%
	N	Y	4.2%	4.5%	7.5%	11.9%	8.3%	8.8%
	Y	N	5.0%	17.1%	20.6%	18.9%	20.8%	20.5%
	Y	Y	5.2%	17.7%	19.9%	19.0%	20.4%	20.5%
Twitter	Y	N	4.7%	16.6%	21.5%	19.9%	21.1%	20.4%
College	N	Y	5.8%	4.1%	18.3%	16.2%	18.3%	17.6%
All Social	Y	Y	5.2%	17.5%	20.4%	19.8%	21.3%	21.2%
All Data	N	N	4.0%	3.1%	4.3%	6.9%	2.2%	5.3%
	N	Y	3.9%	2.6%	4.3%	6.3%	2.5%	4.6%
	Y	N	4.2%	10.3%	19.0%	12.9%	7.6%	18.6%
	Y	Y	4.3%	10.5%	19.0%	14.1%	9.0%	19.3%

S1 Table 23. Summary of algorithm accuracy for 2020 basketball data.

Data Used	Twitter	College	ADA	Logistic Regression	Random Forest	XGBoost	KNN	Extra Trees
Positions Only	N	N	0.006	0.007	0.005	0.006	0.008	0.007
	N	Y	0.022	0.017	0.109	0.106	0.118	0.104
	Y	N	0.024	0.126	0.164	0.148	0.133	0.161
	Y	Y	0.021	0.146	0.17	0.155	0.139	0.164
Team Only	N	N	0.006	0.026	0.029	0.003	0.027	0.025
	N	Y	0.012	0.028	0.036	0.105	0.033	0.044
	Y	N	0.016	0.083	0.169	0.153	0.07	0.174
	Y	Y	0.017	0.084	0.173	0.159	0.07	0.163
Career Length Only	N	N	0.014	0.006	0.041	0.021	0.031	0.034
	N	Y	0.022	0.02	0.164	0.141	0.159	0.16
	Y	N	0.02	0.133	0.157	0.161	0.163	0.168
	Y	Y	0.023	0.129	0.171	0.161	0.16	0.163
Performance Only	N	N	0.009	0.01	0.013	0.021	0.006	0.008
	N	Y	0.016	0.023	0.046	0.05	0.025	0.075
	Y	N	0.021	0.122	0.155	0.122	0.155	0.168
	Y	Y	0.017	0.135	0.157	0.145	0.161	0.17
Rank Only	N	N	0.009	0.003	0.027	0.021	0.026	0.026
	N	Y	0.018	0.012	0.035	0.069	0.027	0.046
	Y	N	0.018	0.128	0.174	0.147	0.168	0.165
	Y	Y	0.019	0.128	0.168	0.149	0.164	0.163
Valuation Only	N	N	0.01	0.004	0.026	0.025	0.027	0.023
	N	Y	0.019	0.019	0.054	0.085	0.066	0.062
	Y	N	0.02	0.132	0.166	0.153	0.168	0.162
	Y	Y	0.021	0.139	0.164	0.151	0.157	0.164
Twitter	Y	N	0.019	0.128	0.175	0.154	0.174	0.162
College	N	Y	0.021	0.007	0.131	0.114	0.128	0.122
All Social	Y	Y	0.021	0.134	0.169	0.157	0.164	0.169
All Data	N	N	0.013	0.026	0.032	0.052	0.015	0.046
	N	Y	0.013	0.021	0.033	0.048	0.017	0.037
	Y	N	0.013	0.084	0.163	0.102	0.058	0.156
	Y	Y	0.015	0.087	0.159	0.112	0.067	0.162

S1 Table 24. Summary of algorithm F1 score for basketball data from 2020.

Data Used	Twitter	College	ADA	Logistic Regression	Random Forest	XGBoost	KNN	Extra Trees
	N	N	(.0, .065)	(.0, .043)	(.0, .0465)	(.0, .055)	(.0, .065)	(.0, .055)
Positions	N	Y	(.0, .12)	(.0, .065)	(.054, .239)	(.043, .229)	(.087, .261)	(.087, .229)
Only	Y	N	(.0, .13)	(.076, .239)	(.097, .294)	(.087, .272)	(.087, .261)	(.087, .294)
	Y	Y	(.0, .13)	(.097, .261)	(.087, .283)	(.109, .283)	(.054, .283)	(.119, .304)
	N	N	(.0, .087)	(.0, .077)	(.0, .109)	(.0, .077)	(.0, .087)	(.0, .065)
Team	N	Y	(.0, .109)	(.0, .077)	(.0, .109)	(.065, .239)	(.0, .087)	(.0, .109)
Only	Y	N	(.0, .87)	(.022, .174)	(.109, .304)	(.097, .304)	(.032, .152)	(.13, .304)
	Y	Y	(.0, .109)	(.043, .174)	(.109, .304)	(.109, .316)	(.01, .164)	(.109, .294)
	N	N	(.0, .087)	(.0, .077)	(.022, .13)	(.0, .087)	(.0, .13)	(.022, .13)
Career	N	Y	(.0, .109)	(.0, .087)	(.097, .316)	(.097, .326)	(.109, .294)	(.109, .283)
Length Only	Y	N	(.0 .13)	(.087, .261)	(.109, .283)	(.119, .304)	(.109, .304)	(.109, .305)
	Y	Y	(.0, .12)	(.087, .239)	(.109, .304)	(.097, .304)	(.13, .283)	(.119, .316)
	N	N	(.0, .065)	(.0, .065)	(.0, .055)	(.0, .065)	(.0, .043)	(.0, .043)
Performance	N	Y	(.0, .109)	(.0, .087)	(.022, .109)	(.01, .142)	(.0, .087)	(.043, .174)
Only	Y	N	(.0, .109)	(.054, .272)	(.109, .283)	(.065, .261)	(.109, .272)	(.097, .326)
	Y	Y	(.0, .087)	(.087, .261)	(.087, .294)	(.109, .272)	(.13, .294)	(.13, .294)
	N	N	(.0, .077)	(.0, .065)	(.0, .087)	(.0, .065)	(.0, .065)	(.0, .065)
Rank	N	Y	(.0, .087)	(.0, .087)	(.0, .098)	(.022, .174)	(.0, .087)	(.022, .109)
Only	Y	N	(.0, .109)	(.076, .239)	(.13, .304)	(.087, .272)	(.109, .304)	(.109, .294)
	Y	Y	(.0, .109)	(.087, .261)	(.13, .304)	(.097, .294)	(.13, .294)	(.119, .304)
	N	N	(.0, .098)	(.0, .065)	(.0, .087)	(.0, .087)	(.0, .065)	(.0, .087)
Valuation	N	Y	(.0, .109)	(.0, .098)	(.022, .152)	(.043, .196)	(.043, .152)	(.043, .142)
Only	Y	N	(.0, .12)	(.097, .272)	(.119, .326)	(.076, .283)	(.13, .304)	(.087, .294)
	Y	Y	(.0, .109)	(.109, .283)	(.109, .304)	(.076, .304)	(.109, .305)	(.119, .316)
Twitter	Y	N	(.0, .109)	(.087, .261)	(.13, .304)	(.097, .283)	(.119, .304)	(.13, .304)
College	N	Y	(.0, .142)	(.0, .087)	(.097, .294)	(.065, .261)	(.109, .283)	(.097, .272)
All Social	Y	Y	(.0, .13)	(.065, .272)	(.13, .316)	(.109, .326)	(.13, .283)	(.097, .272)
	N	N	(.0, .109)	(.0, .087)	(.0, .109)	(.022, .142)	(.0, .065)	(.0, .109)
All	N	Y	(.0, .087)	(.0, .087)	(.0, .087)	(.022, .13)	(.0, .087)	(.0, .109)
Data	Y	N	(.0, .109)	(.043, .174)	(.097, .283)	(.054, .217)	(.022, .142)	(.109, .283)
	Y	Y	(.0, .109)	(.054, .174)	(.109, .294)	(.065, .229)	(.043, .164)	(.109, .272)

S1 Table 25. Summary of 95% confidence intervals for the basketball data from 2020. Each algorithm was run 100 times.

Data Used	College	ADA	Logistic Regression	Random Forest	XGBoost	KNN	Extra Trees
Positions	N	6.1%	11.2%	16.6%	13.4%	13.3%	16.6%
Only	Y	6.0%	11.2%	20.4%	14.0%	17.8%	20.8%
Team	N	6.6%	10.9%	15.8%	12.5%	7.1%	15.4%
Only	Y	6.7%	11.1%	17.7%	13.1%	9.7%	17.3%
Career	N	6.0%	11.9%	16.9%	14.3%	13.6%	16.9%
Length Only	Y	6.0%	12.3%	22.6%	14.7%	20.7%	22.7%
Performance	N	5.9%	11.2%	15.8%	11.7%	12.9%	16.4%
Only	Y	6.0 %	11.3%	16.8%	12.0%	13.5%	17.8%
Rank	N	6.6%	11.0%	15.7%	12.3%	12.8%	16.5%
Only	Y	6.4%	11.2%	16.5%	13.1%	13.7%	16.8%
Valuation	N	5.9%	11.7%	16.5%	12.6%	14.1%	16.9%
Only	Y	6.0%	11.5%	18.7%	13.4%	16.5%	19.9%
College	N	4.3%	4.2%	8.4%	6.8%	7.2%	8.6%
All	N	6.4%	11.1%	16.6%	12.3%	8.7%	17.2%
Data	Y	6.3%	11.3%	18.1%	12.5%	9.0%	18.1%

S1 Table 26. Summary of algorithm accuracy for college basketball data for 2001-2019. As with baseball, the inclusion of social data greatly increases the accuracy, sometimes by over 25%.

Data Used	College	ADA	Logistic Regression	Random Forest	XGBoost	KNN	Extra Trees
Positions	N	0.036	0.118	0.191	0.154	0.147	0.189
Only	Y	0.035	0.117	0.208	0.152	0.175	0.208
Team	N	0.041	0.108	0.17	0.132	0.071	0.164
Only	Y	0.043	0.11	0.179	0.136	0.097	0.173
Career	N	0.033	0.128	0.188	0.158	0.144	0.186
Length Only	Y	0.033	0.127	0.227	0.157	0.206	0.227
Performance	N	0.033	0.127	0.227	0.157	0.206	0.227
Only	Y	0.036	0.12	0.179	0.128	0.136	0.181
Rank	N	0.047	0.123	0.176	0.138	0.134	0.177
Only	Y	0.043	0.121	0.169	0.141	0.137	0.171
Valuation	N	0.035	0.127	0.176	0.141	0.144	0.177
Only	Y	0.035	0.124	0.191	0.145	0.163	0.2
College	N	0.008	0.007	0.074	0.057	0.067	0.077
All	N	0.04	0.111	0.168	0.127	0.086	0.173
Data	Y	0.039	0.113	0.183	0.129	0.089	0.182

S1 Table 27. Summary of algorithm F1 score for college basketball data from 2001-2019. The model with the greatest accuracy also had the highest F1 score.

Data Used	College	ADA	Logistic Regression	Random Forest	XGBoost	KNN	Extra Trees
Positions	N	(.046, .075)	(.094, .127)	(.15, .19)	(.114, .154)	(.117, .156)	(.149, .186)
Only	Y	(.045, .076)	(.094, .13)	(.183, .222)	(.124, .16)	(.159, .2)	(.189, .227)
Team	N	(.049, .083)	(.094, .123)	(.139, .174)	(.107, .142)	(.061, .084)	(.14, .174)
Only	Y	(.051, .084)	(.096, .127)	(.157, .199)	(.116, .147)	(.08, .112)	(.158, .191)
Career	N	(.046, .076)	(.104, .132)	(.147, .188)	(.128, .164)	(.116, .155)	(.149, .187)
Length Only	Y	(.044 .075)	(.11, .139)	(.207, .245)	(.131, .161)	(.186, .227)	(.205, .248)
Performance	N	(.046, .076)	(.095, .129)	(.138, .179)	(.101, .133)	(.112, .147)	(.149, .181)
Only	Y	(.047, .074)	(.099, .13)	(.15, .186)	(.106, .135)	(.115, .152)	(.156, .199)
Rank	N	(.05, .08)	(.095, .126)	(.139, .177)	(.107, .143)	(.11, .142)	(.142, .183)
Only	Y	(.049, .079)	(.096, .127)	(.144, .183)	(.11, .151)	(.117, .155)	(.149, .186)
Valuation	N	(.041, .074)	(.104, .137)	(.149, .181)	(.11, .143)	(.124, .159)	(.148, .187)
Only	Y	(.042, .075)	(.098, .13)	(.167, .211)	(.118, .151)	(.148, .183)	(.174, .218)
College	N	(.032, .053)	(.027, .056)	(.069, .097)	(.053, .082)	(.054, .085)	(.073, .102)
All	N	(.051, .081)	(.095, .129)	(.142, .185)	(.108, .139)	(.074, .101)	(.151, .192)
Data	Y	(.047, .077)	(.099, .128)	(.161, .203)	(.109, .142)	(.078, .102)	(.162, .2)

S1 Table 28. Summary of 95% **confidence intervals for the college basketball data from 2001-2019.** Each algorithm was run 100 times.

Top Attended Colleges	Num of Total Alumni	Max Alumni in a Year	Max Alumni on a Team
Kentucky	119	13	2
UNC	115	11	3
UCLA	98	8	2
UConn	96	9	2
Duke	92	10	2

S1 Table 29. Statistics about the number of alumni from a college playing for the top five schools with the most alumni.

Algorithm	Hyperparameter	MLB Value	NBA Value
Logistic Regression	fit_intercept	False	False
	max_iter	1000	1000
	penalty	none	l2
	solver	newton-cg	liblinear
Ada	learning_rate	1	1
	algorithm	SAMME	SAMME
	n_estimators	75	75
Random	criterion	gini	gini
	min_samples_leaf	1	1
	min_samples_split	4	2
	n_estimators	100	100
	max_features	auto	auto
	max_leaf_nodes	null	None
	max_depth	30	30
KNN	algorithm	auto	auto
	n_neighbors	8	5
	leaf_size	15	15
	weight	distance	distance
Extra Trees	criterion	gini	gini
	max_depth	50	50
	max_leaf_nodes	null	null
	max_features	auto	auto
	min_samples_leaf	1	1
	min_samples_split	6	4
	n_estimators	30	100
XGB	max_depth	3	3
	learning_rate	.1	.1
	min_child_weight	1	1
	reg_alpha	.2	0

S1 Table 30. The hyperparameters used for MLB and NBA models.
